# 24 Hours in the Life of HIV-1 in a T Cell Line

**DOI:** 10.1371/journal.ppat.1003161

**Published:** 2013-01-31

**Authors:** Pejman Mohammadi, Sébastien Desfarges, István Bartha, Beda Joos, Nadine Zangger, Miguel Muñoz, Huldrych F. Günthard, Niko Beerenwinkel, Amalio Telenti, Angela Ciuffi

**Affiliations:** 1 Department of Biosystems Science and Engineering, ETH Zurich, Basel, Switzerland; 2 SIB Swiss Institute of Bioinformatics, Basel and Lausanne, Switzerland; 3 Institute of Microbiology, Centre Hospitalier Universitaire Vaudois, Lausanne, Switzerland; 4 Division of Infectious Diseases and Hospital Epidemiology, University Hospital Zurich, University of Zurich, Zurich, Switzerland; 5 University of Lausanne, Lausanne, Switzerland; Fred Hutchinson Cancer Research Center, United States of America

## Abstract

HIV-1 infects CD4+ T cells and completes its replication cycle in approximately 24 hours. We employed repeated measurements in a standardized cell system and rigorous mathematical modeling to characterize the emergence of the viral replication intermediates and their impact on the cellular transcriptional response with high temporal resolution. We observed 7,991 (73%) of the 10,958 expressed genes to be modulated in concordance with key steps of viral replication. Fifty-two percent of the overall variability in the host transcriptome was explained by linear regression on the viral life cycle. This profound perturbation of cellular physiology was investigated in the light of several regulatory mechanisms, including transcription factors, miRNAs, host-pathogen interaction, and proviral integration. Key features were validated in primary CD4+ T cells, and with viral constructs using alternative entry strategies. We propose a model of early massive cellular shutdown and progressive upregulation of the cellular machinery to complete the viral life cycle.

## Author Summary

Viral pathogens, such as HIV-1, are fully dependent of the cellular machinery to complete the replication cycle. The cell offers a permissive environment, and deploys a number of antiviral defense strategies. The present work follows the process of infection of the cell with simultaneous measurements of viral replication intermediates together with the concurrent assessment of the host transcriptional changes. The main observation is that the cell undergoes a profound modification of its physiology, with a marked early decrease in expression of several thousands of genes, followed by a more discrete increase in the expression of sets of genes that may contribute to the success of the viral replication program. The cell system used in this study has limited response of paradigmatic cellular defense genes. Key features of the experimental model were validated in primary cells and with different viral vectors. The data and model generated here constitute a resource that can be used for the assessment of single gene responses to HIV-1 infection, and as comparative reference for the understanding of other viral and cellular programs, such as those implicated in successful defense against viral infection or in latency.

## Introduction

The life cycle of HIV-1 and its interaction with the host cell has been extensively studied [1]. However, previous analyses did not assess all relevant steps of viral replication in a longitudinal study in a single experimental system. Transcriptome (and miRNA) analyses have used microarray technology, usually in cross-sectional experiments, generally at the completion of the viral replication cycle (24–48 hours) [2]. Analyses of viral integration first evaluated how the transcriptional status of genes contributes to preferential integration of proviruses [3]; however, there is limited data on how the viral integration contributes to host transcription at genome-wide level. Analyses have also been hampered by the heterogeneity of the infectious system, where the transcriptome profile reflects contribution by infected and uninfected cells. Recent studies have approached this problem by magnetic sorting of cells infected in vitro identified by a marker recombinant protein that is expressed during the late-phase of viral replication cycle [4].

Here, we jointly investigated, through repeated measurements in time, the dynamics of viral products and cellular responses in a model of universal cell infection (**Figure S1 in Text S1**). To this end, we applied high-throughput sequencing technologies for mRNA, small RNAs, and viral integration site profiling, as well as detailed quantification of viral replication intermediates. A highly permissive T cell line (SupT1) was chosen, because it could be transduced at 100% efficacy with an HIV vector (NL4-3Δenv::eGFP, VSV.G pseudotyped). This model system allowed effective synchronization through infection and avoided confounding of transcriptional profiles by uninfected bystander cells. Transcriptome changes were shown to be specific to the infectious process, and representative results were subsequently validated using different infection rates, primary cells and alternative viral constructs.

The aim of this project was to create a first model of the productively infected cell by capturing the dynamics of all expressed host genes, concomitantly with viral replication steps. Integration of the cellular and viral data was achieved through rigorous mathematical approaches. The analyses underscored the features of the successful viral replication occurring despite a profound perturbation of the cell at the transcriptional level. Data are provided as a fully interactive web resource to allow reader-specific queries.

## Results/Discussion

### Dynamic analysis of viral replication intermediates

**Figure 1:**
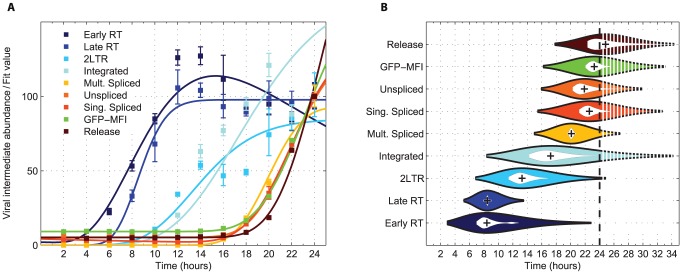
Modeling of the viral life cycle. (**A**) Raw data of measured viral replication intermediates (mean [dots] with one standard error) and curves of fitted progression model (solid lines). The temporal dynamics of each step in the viral life cycle was generated individually by modeling the net effect of production, decay, initial viral input, and experimental noise of the corresponding marker intermediate (**Text S1** and **Figure S4** and **S5 in Text S1**). (**B**) Activity profile of individual steps of the viral life cycle estimated from the progression model. Each violin spans the 98% quantile of the viral step with width proportional to activity level at each given point in time. The plus symbol (‘+’) denotes the peak of the activity and the inner white violin its 95% bootstrap confidence interval. In the shaded area, expected values extrapolated beyond the last observed time point (24 h, dashed line) are shown.

**Figure 2:**
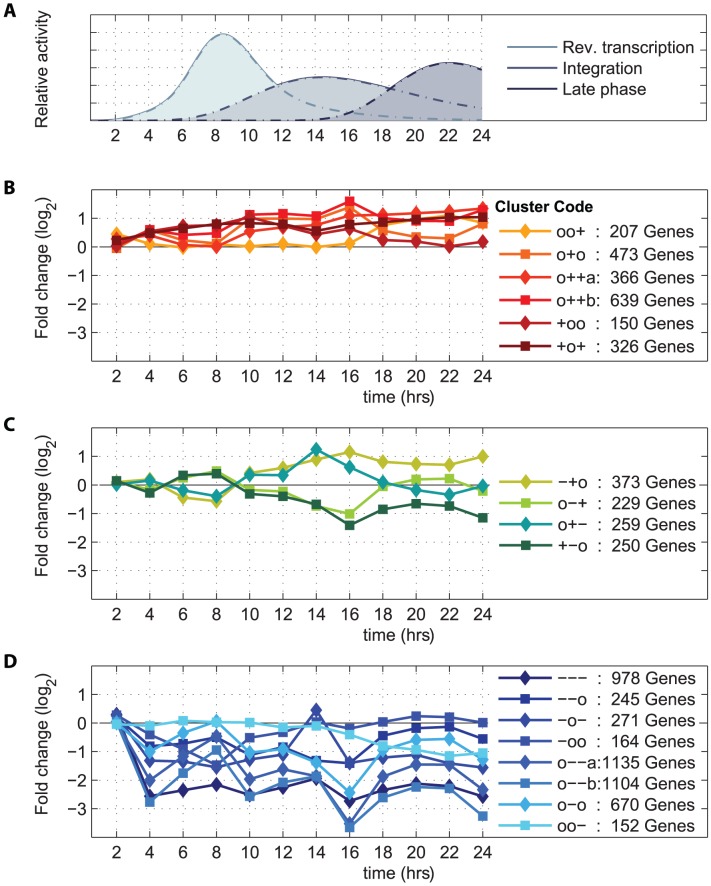
Clusters of host genes correlated with viral progression. Temporal expression patterns of 7,991 genes modulated in concordance with key steps of viral replication (panel **A**) were grouped into 18 clusters with differential expression profiles at three phases of the viral life cycle, namely reverse transcription, integration, and late phase. The cluster code characters ‘+’ and ‘−’ mark significant (*p*<10^−2^) upregulation and downregulation, respectively, while ‘o’ indicates no significant deviation from zero. For example, the cluster ‘−+o’ contains 373 genes downregulated during reverse transcription, upregulated during integration, and unregulated during the late phase. In total, six upregulated clusters (**B**), four clusters with mixed patterns of regulation (**C**), and eight downregulated clusters (**D**) were found. Details of clusters are available at the dedicated web resource [6].

Progression of the viral life cycle was characterized through quantitative measurement of nine species of viral intermediates (**Figure 1A**, and **Figure S2 in Text S1**). To generate a high-resolution picture of the various steps of the viral life cycle, we developed a parametric viral progression model based on ordinary differential equations. We found that initiation of viral reverse transcription (defined as reaching 1% of its total progression) occurs as early as 3 hours after infection, with double-stranded viral cDNA appearing 2 hours later (referred hereafter as *reverse transcription phase*). 2-LTR circles began accumulating as early as 7 hours post-infection, with integration beginning 1.5 hours later (*integration phase*). All viral transcripts emerged by 15 hours and, at the peak of expression, 0.6% of all transcripts in the cell were of viral origin, consistent with previous estimates [5]. Transcription was tightly coupled with translation, and it was followed by the release of viral particles starting at 18 hours after infection (*late phase*) (**Figure 1B**). The temporal patterns of these features of the viral life cycle were used to explain the host genome-wide expression dynamics in response to the invading virus (**Figure 2A**).

### Host transcriptome changes involve a large proportion of cellular genes in concordance with viral progression

High-throughput RNA sequence analysis identified 10,559 genes and 399 miRNAs expressed in the experimental system. Fifty-two percent of the overall variability in the transcriptome was explained by linear regression on the three main phases of the viral life cycle as identified by the viral progression modeling, namely reverse transcription, integration, and late phase (**Figure 2**). 73% of all expressed genes (*n* = 7,991) demonstrated significant correspondence of their temporal expression patterns with steps of the viral life cycle at the 5% false discovery rate. Using the regression weights as a measure of regulation of a gene in each of the three viral life phases defined above, we found 18 co-regulated gene clusters (**Figure 2**, and **Figure S7** and **S8 in Text S1**). Clusters were assessed for enrichment in gene ontology terms and pathways. Detailed inspection of clusters, individual querying of genes and of gene sets is provided at a dedicated web resource (www.peachi.labtelenti.org [6]).

Downregulation of cellular genes was generally early (4 hours post-infection), profound, and persistent throughout the experiment (**Figure 2**, and **Figure S8C in Text S1**). The eight downregulated clusters, including 4,719 (43%) genes, coherently exhibited enrichment in several functional gene sets. For example, downregulation concerned 70% (538/751, *p*<10^−13^) of all expressed genes encoding nuclear proteins, 70% (338/484, *p*<10^−6^) of genes involved in the Reactome expression machinery, such as those encoding RNA polymerase II components, splicing factors, ribosomal proteins, tRNA synthetases and translation initiation factors, and 75% (185/248, *p*<10^−7^) of genes involved in protein metabolism. Despite the observed cellular response to infection, cell viability was similar in mock and infected cells (75% vs 72% at 24 hours, respectively). The observed pattern of cellular shutdown is more consistent with a cellular response to viral invasion than with experimental stress, given that two hours after infection, the transcriptome of HIV-infected cells is undistinguishable from that of mock samples.

In contrast to the downregulated genes, upregulation occurred progressively and at later time points (**Figure 2**, and **Figure S8A in Text S1**). Six clusters containing a total of 2,161 (20%) genes were upregulated in response to infection. Overrepresented gene groups notably identified the Reactome generic transcription pathway (43%, 47/108, *p*<10^−4^) that includes components of the mediator complex and zinc finger proteins. Individual upregulated clusters showed overrepresentation of several signaling and innate immune pathways, such as cytokine-cytokine receptor interaction (*p*<10^−3^), TLR signaling (*p* = 0.0016), and activation of NF-κB (*p*<10^−3^). Genes involved in antiviral defense and cell death signaling were also enriched in the four clusters, comprising 1,111 (10%) of the genes, that exhibited mixed patterns of upregulation and downregulation (**Figure 2**, and **Figure S8B in Text S1**). Thus, the early, large-scale, coordinated shutdown of the cell was followed by upregulation of immune response signals suggesting the triggering of defense mechanisms by the cell. However, detailed analysis of selected mechanisms of antiviral defense portrayed the extent to which the highly permissive cell line used in the current study may be poorly equipped to respond to the incoming virus. For example, of 331 interferon stimulated genes previously tested against HIV-1 [7], less than half (n = 144, 43%) were expressed in SupT1 cells, and only 61 (18%) were upregulated in concordance with the viral life cycle. In particular, among the 6 most active anti-HIV interferon stimulated genes described before [7], most were not expressed and only *IRF1* was upregulated. Similarly, of the four prototypical lentiviral restriction factors, *TRIM5α*, *APOBEC3G*, *BST2*/*Tetherin*, and *SAMHD1*, only *TRIM5α* was expressed and upregulated. The paucity of innate immunity gene expression may contribute to the high permissiveness of SupT1 T cells to infection, and thus, to their frequent use of in HIV-1 research.

### Many transcripts coding for HIV-interacting proteins are downregulated upon infection

We further examined the pattern of expression of host genes reported to interact with HIV-1 proteins. We first analyzed the expression profile of 443 genes previously identified in a screen of physical interactions of all 18 HIV-1 proteins with human factors [8]. Of these, 382 were expressed in our experimental system, and 290 (76%) showed modulation associated with viral progression features; specifically, 55% downregulation, 13% upregulation, and 8% mixed regulation. The enrichment of virus interaction partner genes was significantly higher in down-regulated clusters as compared to overall cellular transcripts (43%, 20%, and 10%, respectively; *p*<10^−6^). Specific clusters were enriched with genes encoding interaction partners of the viral proteins Vif, gp41, Vpr, and Tat. Additional databases of HIV-1 host factors [9], [10], [11], [12], [13], as well as genes present in HIV-related pathways extracted from Reactome were inspected in a similar fashion [6]. Most of them were downregulated, emphasizing the importance of assessing interactions between viral and host genes in the context of the dynamics of the infection process and not as static events.

### Transcriptome analysis reveals correlation for transcription factors and their targets but not for miRNA and cognate targets

Transcription factors and miRNAs are two key components of transcriptional regulation. Over two thirds of the 18 co-regulated gene clusters exhibited significant overrepresentation of the putative targets of one or more transcription factor or miRNA. Several major transcription factor genes were downregulated along with their corresponding targets. For example, 1,080 (23%) of the downregulated genes were targets of the large-scale transcriptional regulators SP1, MAZ, and ELK1, that were found also to be downregulated (**Table S1 in Text S1**). In contrast, there was limited agreement between miRNA expression and that of genes sharing the experimentally verified miRNA targets (**Table S2 in Text S1**). Specifically to HIV-1, only miRNAs that target the viral 3′LTR (miRNA-125b, miRNA-150, and miRNA28-3p) and experimentally shown to inhibit HIV-1 [14], were found to be upregulated during the infection [6]. These results underscore the difficulty in interpreting regulator-to-regulated gene activities in complex settings such as the infected cell.

### Viral integration events in genes do not account for transcriptome changes at the population-level

**Figure 3:**
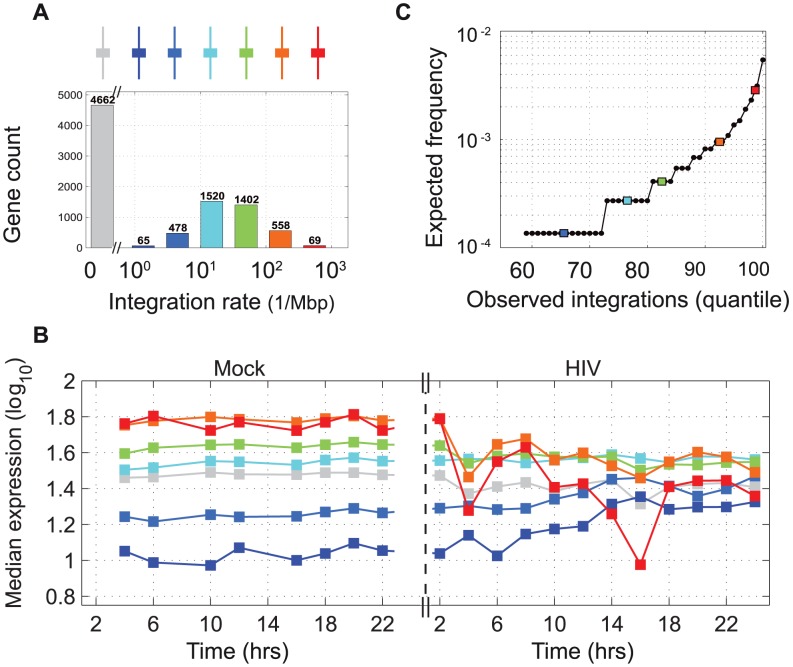
Host gene expression and viral integration. (**A**) Distribution of observed rates of integration: six gene groups (color coded) were defined based on the number of integrations per megabasepair (Mbp). Genes with no observed integration are depicted in grey. (**B**) Average level of expression of the six gene groups in Mock and HIV-infected cells during the 24-hour experiment. (**C**) Expected number of integration events in individual genes based on an empiric integration rate of 5.5 proviruses per haploid genome and on 40,430 observed unique integration sites (approx. 1% of all events).

Many chromosomal regions were enriched in gene clusters, suggesting location-specific co-regulation. We investigated the possibility that such regional gene expression profiles are influenced by the spatial pattern of HIV-1 integration into the host genome. We identified 40,430 unique viral integration sites. Consistent with previous work [15], integration favored genes that are transcriptionally active prior to infection, and this association remained at the time of integration, although many genes had, by then, undergone significant downregulation (**Figure 3**). At 24 hours there was a negative correlation of −0.26 (*p*<10^−64^) between the frequency of integration in a given gene and the observed change in gene expression. However, given the low prevalence of integrations in the overall cell population (**Figure 3**), even genes with the highest number of integration events were unlikely to be downregulated by more than 0.008 *log_2_* fold at the cell population level (**Figure S9 in Text S1**). Thus, while cellular gene expression levels influenced integration rates, proviruses did not contribute significantly to global cellular expression levels. This observation does, however, not preclude an impact of integration at the single cell level.

### Exposure to non-infectious viral material does not explain the observed transcriptome changes

**Figure 4:**
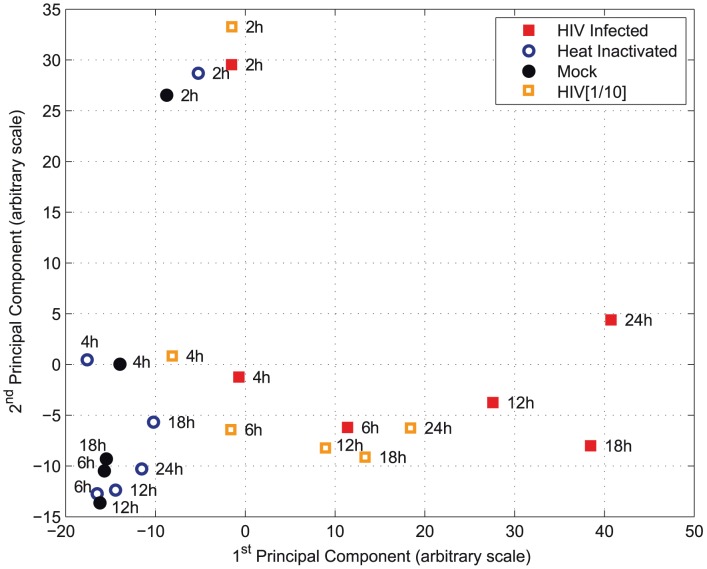
Transcriptome changes upon exposure to infectious and non-infectious viral particles. Principal component analysis is used to explore the overall variance structure of the transcriptome datasets. With each point representing a whole transcriptome sample, the figure presents the transcriptome of cells that were universally infected (HIV), cells exposed to heat-inactivated virus (Heat-inactivated), cells exposed to a mixture of 1∶10 infectious/heat-inactivated virus (HIV[1/10]), and non-infected cells (Mock). One mock sample failed and is not plotted. The transcriptome of mock cells and that of cells exposed to heat-inactivated viruses clustered together across the top principal components. Infected cells, on the other hand, spread away from the mock space as infection progressed, with the most distant dot corresponding to the latest time point (24 h). The mixture 1/10 infectious/noninfectious material occupies the intermediate space. Clustering of the two hours samples corresponds to end of cell exposure to the virus or control materials.

One of the difficulties in trying to study HIV infection in cultured cells, as compared with what may happen *in vivo*, is the use of a large multiplicity of infection, and the exposure of the cells to large concentration of non-infectious particles. To assess the possibility that the profound transcriptome modifications were due to exposure to non-infectious viral particles, we compared the transcriptome of cells that were universally infected, cells exposed to heat-inactivated virus, cells exposed to a mixture of 1∶10 infectious/heat-inactivated virus, and non-infected (mock) cells. The transcriptome of mock cells and after exposure to heat-inactivated viruses clustered together across the top principal components (**Figure 4**). Infected cells spread away from the mock space as infection progressed. These data confirm that the transcriptome changes reflect the viral progression and is not a mere result of exposure to viral material.

### HIV-mediated gene regulation is recapitulated using different viral and cellular systems

**Figure 5:**
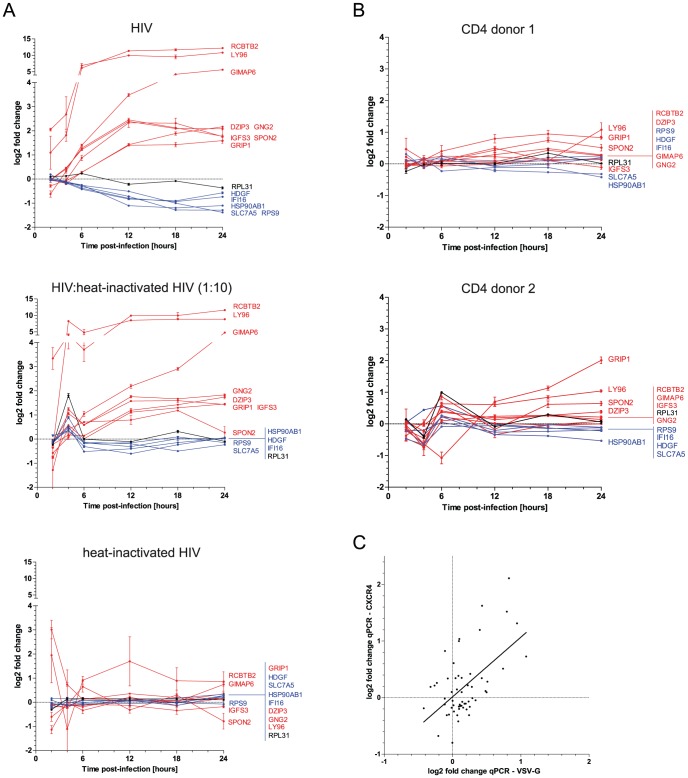
Core gene validation. RT-qPCR was used to validate key patterns of expression using heat-inactivated virus, primary cells, and natural viral envelope. (**A**) Analysis of 14 representative genes using competent or heat-inactivated HIV-based vector. The graphs depict the 24 dynamics of expression (log_2_ fold change of VSV.G pseudotyped HIV-infected over mock) of eight upregulated genes (red lines), five downregulated genes (blue), and one control (*RPL31*, black line) in SupT1 cells exposed to similar amount of viral particles, only competent HIV (top panel), 1∶10 competent HIV∶heat-inactivated HIV (middle panel), and only heat-inactivated HIV (bottom panel). (**B**) Analysis in primary CD4+ T cells isolated from two healthy blood donors. Depicted are the 24 dynamics of expression (log_2_ fold change of VSV.G pseudotyped HIV-infected over mock) of the upregulated (red), downregulated (blue), and control (black) genes. (**C**) Correlation analysis of RT-qPCR for the 14 representative genes at all time points in primary cells (donor 1) infected by VSV.G or CXCR4 pseudotyped HIV. Log_2_ fold change linear regression yielded *r^2^* = 0.22, *p*<10^−4^.

The experimental system consisted of a highly permissive T cell line (SupT1) and a VSV.G pseudotyped HIV vector to achieve universal infection. To validate our results, we used primary cells and natural viral entry. Activated CD4+ T cells from two donors were transduced with HIV vectors pseudotyped with both VSV.G and CXCR4-tropic envelopes. As expected, the rate of infection of primary cells was inferior to that of the T cell line (**Figure S10 in Text S1**). We analyzed the expression of 14 genes representative of various clusters by RT-qPCR. First, we compared the gene expression findings based on RNA sequencing with RT-qPCR data. For example, at 24 hours after infection, the correlation between the two techniques was *r* ^2^ = 0.77 (*p*<10^−4^) even though the dynamic range is larger for RNA sequencing (**Figure S11 in Text S1**). Second, we re-assessed the role of exposure to non-infectious viral material in modifying expression of the marker genes (**Figure 5A**).We also applied RT-qPCR to the analysis of gene expression patterns over the 24-hour viral life cycle in primary cells (**Figure 5B**). Finally, we compared transduction of primary cells by HIV-1 carrying natural (CXCR4) with vectors pseudotyped with VSV.G envelope (**Figure 5C**). Overall, genes upregulated in SupT1 cells were generally confirmed as upregulated in primary cells, but the signal was weaker in primary cells due to dilution by RNA of non-infected bystander cells and possibly by cell-specific responses to HIV-1. Downregulation was muted in primary cells despite equal experimental conditions, including biological stress, indicating that cellular shutdown is a response to successful infection. In support of this notion, a lower infection rate (1/10 inoculum, diluted in heat-inactivated virus preparation) resulted in proportional modulation (up- or down regulation) of the signal; and cells exposed to 100% heat-inactivated virus were comparable in expression pattern to mock-treated cells.

### Conclusions

Research on the infected cell generally follows the paradigm of “single gene, single interaction”. However, this approach fails at fully capturing and quantifying the complexity of the system. In contrast, the non-reductionist study presented here reflects the intricate cellular response to infection where, at the transcription level, a large proportion of genes are modulated in concordance with key steps of viral replication. As such, this work provides a referential resource on the viral life cycle that can be contrasted across cellular systems and viral strains, and also across diverse pathogens. The approach should be extended to study the establishment of and reactivation from viral latency [16]. Ultimately, it can guide intervention of the viral life cycle at specific time points through the modulation of selected host genes and pathways. Progress in single-cell transcriptome analysis should allow in the future to investigate primary cells infected with replication-competent virus.

## Materials and Methods

### Viral life cycle

#### Virus production

HEK293T cells were co-transfected with 15 µg pNL4-3ΔEnv-GFP (NIH AIDS Research and Reference Reagent program, Cat. #11100) and 5 µg pMD.G, using the calcium phosphate method (Invitrogen). pNL4-3ΔEnv/GFP encodes the HIV vector segment with a 903 bp deletion in the *env* ORF in which the *gfp* ORF was introduced [17]. The second plasmid pMD.G codes for the vesicular stomatitis virus G envelope (VSV-G) [18]. Forty-eight hours after transfection, the supernatants were collected, centrifuged, and filtered through 0.45-µm filters. Viral particles were concentrated by filtration on Centricon units (Centricon Plus-70/100K, Millipore). The concentrated viral supernatant was treated with 100 units/ml DNase I (Roche) for 1 h at 37°C and stored at −80°C. Viral titers were measured by p24 ELISA (Abbott Murex).

#### Cellular infection and sample collection

SupT1 cells (5×10^6^ cells) were either mock treated or infected with 15 µg p24 equivalent of HIV-based vector by spinoculation at 1500 g for 30 min at room temperature, in presence of 5 µg/ml polybrene (Sigma), in 400 µl final volume – for a total of 72 tubes for mock and 72 tubes for infected condition (**Figure S1 in Text S1**). After three washes with culture medium, cells were pooled, resuspended at 10^6^ cells/ml in R-10 and further incubated. Every two hours, cellular samples (∼30×10^6^ cells in 30 ml) were collected for viral and cellular measurements. Briefly, 0.5 ml of the cell culture was used for cell counting and viability assessment by trypan blue exclusion, using a ViCell Coulter Counter (Beckman Coulter). Remaining cells were centrifuged at 300 g for 10 min. On one hand, 950 µl supernatant was collected, mixed with 50 µl NP-40 and stored at −80°C until particle release assessment by p24 ELISA (Abbott Murex). On the other hand, cells were washed with PBS once, centrifuged again, resuspended in 3 ml PBS (∼10^7^ cells/ml) and separated as follows: (i) 50 µl of cell suspension were resuspended in Cell Fix 1× (Becton Dickinson) for assessment of GFP expression by FACS analysis (FACSCalibur, Becton Dickinson), (ii) 300 µl of cell suspension were centrifuged and stored at −80°C as a dry pellet for subsequent DNA extraction and viral DNA form analysis, (iii) 1.5 ml of cell suspension were centrifuged, resuspended in 100 µl PBS, complemented with 1 ml RNALater (Ambion) and stored at 4°C for further RNA extraction and gene expression analyses.

#### Reverse transcription and integration

DNA was extracted using DNeasy Blood and Tissue kit (Qiagen), and quantified using Nanodrop-1000 spectophotometer (Nanodrop). Viral DNA forms (early RT, late RT, 2-LTR) were assessed by qPCR as described in [10]. Briefly, 20 ng DNA were mixed with 1 µM forward and reverse primers (**Table S3 in Text S1**), 0.2 µM probe, and 1×Taqman Gene Expression Master Mix (Applied Biosystems) in a final volume of 25 µl. qPCR was carried in triplicate in the StepOnePlus Real-Time PCR system (Applied Biosystems) using standard cycling conditions, *i.e.* 2′ at 50°C, 10′ at 95°C, 40 cycles of 15″ at 95°C and 1′ at 60°C. *HMBS* (*PBGD*) was used as the endogenous control. The comparative C_T_ method was used for relative quantification, *i.e.* to assess fold change calculations using the 24 h time point as the reference, and according to the 2^−ΔΔCT^ formula (Guide to Performing Relative Quantitation of Gene Expression Using Real-Time Quantitative PCR, section VII.3, Applied Biosystems). To quantify the viral integrated DNA products, a first Alu-gag PCR was carried out using 20 ng DNA, 0.4 µM primers (**Table S3 in Text S1**), and Accuprime Pfx Supermix (Life Technologies) in a 25 µl final volume reaction. PCR cycling conditions were 5′ at 95°C, followed by 25 cycles of 30″ at 95°C, 15″ at 55°C, 4′ at 68°C, and finally 10′ at 68°C. One tenth of this first PCR was used for qPCR as described above.

#### Viral transcription

Cell samples were stored in RNALater at 4°C until RNA extraction with Trizol Reagent (Life Technologies). Total RNA was quantified using Nanodrop-1000 spectrophotometer (Nanodrop) and Total RNA Nanochip (Agilent). Viral splice variants were assessed by one-step RT-qPCR (Qiagen) in duplicate using different pairs of primers and probe (**Table S3 in Text S1**), essentially as described in [19]. Briefly, a lower-phase mix containing 10 µl of 1× One-Step RT-PCR buffer, 0.5 mM MgCl_2_, 1 µM forward and reverse primers, 0.3 µM probe was topped with 15 µl Ampliwax (Applied Biosystems) and sealed for 5′ at 90°C, and thus separated from the top-phase mix containing 20 µl with 1× One-Step RT-PCR buffer, 0.2 µM reverse primer, 0.4 mM each dNTP, one-step RT-PCR enzyme mix and 1 µl of total RNA. cDNA synthesis was carried out in a real-time IQ5 thermocycler (BioRad) for 30′ at 50°C, immediately followed by qPCR with the following cycling conditions: 15′ at 95°C, followed by 50 cycles of 5″ at 95°C and 40″ at 60°C. The viral transcripts at 24 h were measured by endpoint dilution. qPCR of the 2 h to 22 h samples was performed by the comparative C_T_ method relative to the 24 h reference time point. GAPDH was used as endogenous control [19].

#### Modeling viral progression

The temporal dynamics of the measured viral life cycle markers were modeled explicitly using an ordinary differential equation. We defined the true (noise-free) abundance of the marker, *x_t_*, as the net effect of production, decay, and initial viral input as
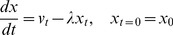
(1)where, *v_t_*, denotes the production rate of the marker (due to the activity of the corresponding step in viral life cycle), and λ is an exponential decay rate accounting for potential loss of the marker over time. The measured marker abundance, *y_t_*, was modeled as the true marker abundance distorted by experimental noise. The decay term λ and initial viral input *x_0_* were assumed to be non-negative. The marker production rate over time, *v_t_*, was assumed to have the shape of a gamma distribution function, which describes the distribution of the waiting times until production of one unit of the marker. The model was fitted to the data using iterative nonlinear least-squares optimization after accounting for the structure of variance-mean dependencies in the used measurement platforms, and the differential equation was solved numerically at each step of the optimization procedure. A parametric bootstrapping scheme was applied to derive confidence intervals of the peak activity at each viral life step. A detailed description of the model construction and fitting procedure is available in Text S1.

### Host transcriptome

#### SAGE library preparation and high-throughput sequencing

Total RNA was extracted using Trizol (Invitrogen). Quality was assessed by capillary electrophoresis using a total RNA NanoChip in the 2100 Bioanalyzer (Agilent). RNA was quantified using Qubit fluorometer (Invitrogen). Gene expression profiles were obtained by generating a SAGE library followed by high-throughput sequencing using SOLiD 3 (Sequencing by Oligonucleotide Ligation and Detection) system technology (Applied Biosystems) [5]. Briefly, polyadenylated RNA from total RNA (3 µg) were captured on oligodT-conjugated magnetic beads and reverse transcribed with the SuperScriptIII reverse transcriptase. cDNA was subsequently digested with NlaIII and ligated to a first adapter that is NlaIII-compatible. A second digestion was performed using Eco15PI that recognizes a sequence in the first adapter and cuts 25 bases away. A second barcoded adapter Eco15PI-compatible was ligated, generating a 27 bp tag fragment surrounded by two adapters, that is transcript- and strand-specific. After DNA purification, sequencing was performed with a universal primer complementary to the first adaptor.

#### Preprocessing of SAGE data

SAGE reads were aligned to the reference genome using Bowtie version 0.12.7 [20]. The reference genome was built using human genome assembly HG37 release 60 along with the HIV-1 genome as an additional chromosome. Three adapter nucleotides were removed from the 3′ ends of the reads prior to alignment. The alignment was performed allowing up to 3 mismatches in a 17 bp-long seed sequence. Reads with multiple alignment hits were randomly assigned to one of the sites with the highest alignment score (Bowtie parameters −M 100 −k 1 —best —strata). Reads with alignment hits starting at, or ending in ±4 nucleotides from an NlaIII recognition site, CATG, were retained for further analysis depending on the chromosomal strand they were aligned to. The last two CATG sites for each transcript were taken into account for annotating SAGE tags in a similar fashion as in the mapping pipeline proposed in [21]. Gene-level expressions were generated by summing the expression values of all their corresponding transcripts. A total of 10,569 genes were called expressed based on at least three reads per one million valid reads in at least two of the samples. Expressions levels were normalized for library size differences using the median fold change as suggested in [22]. The median absolute deviation of log_2_ fold changes in expression was calculated as a robust statistic assessing the dispersion in the samples. Mock samples with a dispersion larger than 0.74 (one median absolute deviation away from the expected dispersion) were excluded from the downstream analysis (2 hr, 8 hr, 14 hr, and 24 hr).

#### Small RNA library preparation and high-throughput sequencing

Total RNA was extracted using Trizol (Life Technologies). Quality was assessed by capillary electrophoresis using a small RNA chip in the 2100 Bioanalyzer (Agilent). Library preparation was performed using the Total RNA-Seq kit (Life Technologies) starting with 2 µg of total RNA and according to manufacturer's instructions. Briefly, total RNA was ligated to adapters, reverse transcribed, purified, size selected on gel, amplified by PCR with barcoded primers, purified and size selected on gel (110–130 bp) again. Emulsion PCR (ePCR) and SOLiD sequencing were performed as described for SAGE samples.

#### Preprocessing miRNA data

Low-quality reads (as identified by the ABI standard protocol) and reads with ambiguous bases were removed. Using the FASTX-Toolkit (http://hannonlab.cshl.edu/fastx_toolkit/index.html), the primer sequence was clipped from the read and identical reads were collapsed. Reads shorter than 13 nucleotides were discarded. Mapping was performed using MegaBLAST [23] with the following parameters: wordsize 8, penalty for mismatch −3, reward for match 1, open gap cost −1, and extend gap cost −1. Mapping was performed on mature human miRNA stored in mirBase release 17 [24]. Mapping results were filtered for percentage identity and coverage over 90%. As a final step, miRNA with average counts below 10 across all samples were discarded.

### Modeling of the host transcriptome

#### Regression analysis

In order to characterize the association between the sequence of viral events and cellular gene expression profiles, we examined the linear correspondence of host gene (including mRNA and miRNAs) expression patterns to the three main phases of the viral life cycle, namely reverse transcription, integration, and late phase. Each of the three columns in the feature matrix, *z*, is the average of the estimated activity of its corresponding markers between the measurement time points. Measurement values from mock and HIV-infected samples were concatenated together and the viral activity was set to zero in the mock samples. An expression pattern vector, *g_i_*, was constructed for each gene *i* in a similar manner by concatenating log_2_ expression levels of the mock and HIV-1 samples. Each gene was modeled individually by the linear regression model
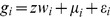
(2)as a function of viral activity, *z*, constant mean estimator *μ_i_*, and a zero-mean Gaussian noise vector *ε_i_*. We used the log transformation of gene expression values as a variance stabilizing transformation that maintains the interpretability of the regression results as proposed for SAGE data in [21]). Each regression coefficient, i.e., each entry in *w_i_*, can be regarded as a measure of the level of regulation of gene *i* by the corresponding HIV-1 life cycle feature. The significance of the fit was evaluated using the standard F-statistic for linear regression, and q-values were computed as the positive false discovery rate (pFDR)-corrected version of the original p-values as described in [25].

#### Clustering of gene expression time courses

Gene expression profiles over the 24 h observation time period were clustered to identify co-regulated gene sets. For this purpose, we analyzed all 7,991 genes that were significantly described by the regression model, i.e., for which at least one regression coefficient in *w_i_* was significantly different from zero, defined by a q-value below 0.05. Clustering was performed on the regression coefficient vectors using the cosine distance and the k-means clustering algorithm [26]. The number of clusters was chosen in a data-driven fashion based on the Bayesian information criterion [27]. A detailed description of the cluster analysis and model selection procedure is available in Text S1.

#### Enrichment analysis

Enrichment analysis was performed using Fisher's exact test based on the hypergeometric distribution to test for over-representation of specific gene sets in the clusters. Enrichment tests were performed in two ways, first for the major regulation groups, namely, upregulated, downregulated, and mixed, and second for each of the 18 gene clusters individually (**Figure S8 in Text S1**). We tested for the presence of the following types of regulation:

“Location-specific”: Genes were labeled according to two separate types of co-localization, one classifying genes by their physical position on the chromosomal bands, and the other according to the Gene Ontology (GO) cellular component classification of the genes. Both annotations are available from the Molecular Signatures Database (MSigDB ver. 3, www.broadinstitute.org/gsea/msigdb) [28].

“Sequence-based”: We checked sequence-based regulations by analyzing sets of genes that share the same transcription factor binding motif as defined in the TRANSFAC database (version 7.4, http://www.gene-regulation.com), and genes sharing experimentally verified miRNA regulation as reported in TarBase 6.0 [29].

“Functional”: We used canonical pathway classification of genes according to the Reactome database (ver. 40, http://www.reactome.org), GO biological process, GO molecular function, and a selected list of canonical pathways included in MSigDB from KEGG pathways (www.genome.jp/kegg/pathway.html) and BioCarta (www.biocarta.com/genes/index.asp).

“HIV-1-related”: We compiled a list of previously reported HIV-1 related genes. This list included HIV-1 host factors reported in [9], [10], [11], [12] as well as the genes classified by the viral protein-protein interaction partner of their corresponding protein product, reported for each viral protein in [8] and in the VirusMINT online database (http://mint.bio.uniroma2.it/virusmint/Welcome.do).

The FDR was controlled according to the procedure in [30], for each database for all the tested clusters simultaneously, and hits below 5% FDR are reported. Results and the used databases are available for download and querying at the online resource [6].

### Viral integration

#### Viral integration site analysis

Identification of viral integration sites in the 24 h time point sample was performed as previously described [31], [32]. Briefly, DNA was extracted from infected cells, digested with MseI or NlaIII, and ligated to a specific compatible linker. The host flanking DNA sequence was amplified by PCR using specific primers annealing to the 3′ LTR and to the linker sequence respectively. A nested PCR was performed using primers with tails that contained a specific barcode and a universal sequence necessary for subsequent high-throughput sequencing using 454 pyrosequencing technology (DNA sequencing facility, University of Pennsylvania). Sequences were analyzed using the InSiPiD program from Frederic Bushman's lab (http://microb215.med.upenn.edu/). Sequences were trimmed from HIV-1 and linker sequences, and aligned with the human genome (hg18) using BLAT. Integration sites were considered to be true if alignment with the human genome started within the first 3 nucleotides, had a >98% sequence identity, and had one single best hit for viral integration positioning.

#### Quantification of integration events per cell

The absolute quantification of integrated HIV-1 copies was done by qPCR essentially as described in [33]. Briefly, viral LTR sequences and *PBGD* human gene were amplified by PCR using primers MA.pr-251 to MA.pr-254, respectively (**Table S3 in Text S1**) and cloned in TOPO TA plasmids. Dilutions of these plasmids carrying different ratios of viral LTR copies and *PBGD* (LTR/PBGD, 1∶1; 1∶2; 1∶3; 2∶1; 0∶1 and 1∶0) were used to assess the ΔCT (C_T_ LTR-C_T_ PBGD) by qPCR allowing generation of a standard curve and calculation of the number of LTR per PBGD copy in our samples. As infected cells may carry multiple forms of viral DNA sequences (linear viral DNA, 1-LTR circles, 2-LTR circles and integrated viral DNA) that may bias quantification, we first established a standard cell curve as reference. For this, SupT1 cells were transduced with an HIV-based vector containing the puromycin resistance gene in the *env* ORF, and selected for two weeks in presence of 1 µg/ml of puromycin (Invivogen), thereby allowing the dilution of non-integrated viral DNA forms and thus removing the bias quantification, *i.e.* the number of LTR copies measured reflects the number of proviruses. LTR∶PBGD ratios from TOPO plasmids (log_2_ transformed) were plotted against ΔC_T_ (C_T_ LTR – C_T_ PBGD) allowing to calculate the LTR∶PBGD ratio in the reference cell line by linear regression, which was 2.75 proviruses per PBGD copy. The standard cell curve was generated by mixing the reference cell line (infected) with uninfected cells at different ratios (100% inf.-0% uninf.; 70% inf. −30% uninf.; 10% inf. −90% uninf. and 0% inf. −100% uninf.). Linear regression of provirus∶PBGD ratios (log_2_ transformed) and ΔC_T_ (C_T_ provirus – C_T_ PBGD) on dilutions of the reference cell line allowed to calculate that the number of proviruses.

#### Estimating population frequency of viral integrations and its impact on expression

Let *N* be the total number of viral integrations in the experiment and *φ_i_* the number of integrations observed within the borders of gene *i*. In general, the intragenic integrations *φ_i_* do not sum up to *N*, because not all integrations fall into gene-coding regions. Let *κ* be the average number of viral integrations per haploid genome in the sample. Then the expected proportion of cells, *ρ_i_*, hosting proviral integrations in gene *i* is
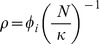
(3)where *N*/κ is the effective number of cells measured in the experiment. Genes were partitioned into 100 quantiles based on the observed number of viral integrations *φ_i_* in them, and the population frequency was calculated for the median of each quantile with *N* = 40,430 and *κ* = 5.5. We employed two extreme and opposing scenarios in order to obtain estimates of the ability of viral integration to impact gene expression at the population level. Given the random nature of proviral integration and the fact that, in practice, *ρ_i_*<<1 for all genes, we assume that no single cell hosts more than one viral integration in a specific gene. Suppose that viral integration affects the transcriptional activity of the gene by a factor of *ζ* irrespective of the exact location or direction. Then the expected change in the log_2_ expression level, *g_i_*, of gene *i* due to integration is
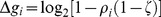
(4)For the first scenario, it is assumed that a single integration event in a gene completely knocks out its transcription (i.e., *ζ* = 0 in **Eq. 4**). For the second scenario, we consider the case of proviral integration boosting transcriptional activity. In this case, we set *ζ* = 105 as measured by a luciferase reporter assay upon transfection of HEK293T cells with an empty luciferase vector compared to a luciferase driven by the HIV-1 LTR (data not shown), which can be regarded as an upper bound of *ζ*.

### Primary cell and natural envelope infection

#### Cells and viral constructs

CD4+ T cells were isolated from two healthy blood donor buffy coats and stimulated using anti-CD3/anti-CD28 and IL-2 as described in [34]. CXCR4-tropic HIV vectors were produced as described for VSV-G pseudotyped HIV vectors, with the following differences: the use of a CXCR4 encoding expression vector (pCI-X4-Env; from R.F. Siliciano [34]) instead of the pMD.G plasmid, transfection was carried out using Lipofectamine 2000 (Life Technologies) and viral particles were purified on sucrose cushion as described [34]. CD4+ T cell transduction by CXCR4-pseudotyped HIV was performed as for HIVeGFP/VSV-G, however with a 3 h spinoculation to improve transduction efficiency.

#### Gene expression assays

Total RNA was extracted using Illustra RNAspin mini isolation kit (GE Healthcare). RNA (2 µg) was reverse transcribed using High-Capacity cDNA Reverse Transcription (Life Technologies). After cDNA purification (Invitek), DNA was quantified using Nanodrop-1000 (Nanodrop) and diluted at 5 ng/µl. Fourteen representative genes from upregulated and downregulated clusters were selected and quantified by qPCR using 10 ng cDNA, and commercially available Gene Expression Assays (Applied Biosystems, **Table S4 in Text S1**). *PIGS* mRNA was used as endogenous control. Calculations were ΔΔC_T_ = (C_T_ gene−C_T_ PIGS)_HIV_−(C_T_ gene−C_T_ PIGS)_mock_. Log_2_ fold change of RT-qPCR data of HIV-1 over mock samples corresponds to the −ΔΔC_T_. For comparison with SAGE-Seq data of HIV-1 samples at individual time points, the read count of each gene was normalized first by *PIGS* and then compared to the mean of mock samples.

## Supporting Information

Text S1 Includes material and methods for modeling viral progression, material and methods for clustering of gene expression time courses, 11 supporting figures and 4 supporting tables.
(PDF)
