# Correction: 24 Hours in the Life of HIV-1 in a T Cell Line

**DOI:** 10.1371/journal.ppat.1005006

**Published:** 2015-06-15

**Authors:** Pejman Mohammadi, Sébastien Desfarges, István Bartha, Beda Joos, Nadine Zangger, Miguel Muñoz, Huldrych F. Günthard, Niko Beerenwinkel, Amalio Telenti, Angela Ciuffi

The article is missing grant details in the Funding section. Please see the correct Funding Statement here:

This work was funded by the Swiss National Science Foundation (grant 310030–130699) to AT, AC and NB, by a Grand Challenges Exploration award from the Bill and Melinda Gates Foundation, and by the European Union's Seventh Framework Programme FP7/2007–2013/ under grant agreement n°305762. HFG is supported by SNF grant 127317. The funders had no role in study design, data collection and analysis, decision to publish, or preparation of the manuscript.
