# Supplementary material for: 24 Hours in the Life of HIV-1 in a T Cell Line
Source: PLoS Pathog. 2013 Jan 31;9(1):e1003161. doi: 10.1371/journal.ppat.1003161 (PMC3561177; doi:10.1371/journal.ppat.1003161)
Supplement: Text S1 — Includes material and methods for modeling viral progression, material and methods for clustering of gene expression time courses, 11 supporting figures and 4 supporting tables. (PDF) [file ppat.1003161.s001.pdf]

Supporting Information, Text S1

24 hours in the life of HIV-1

Pejman Mohammadi, Sébastien Desfarges, Istvan Bartha, Beda Joos, Nadine Zangger, Miguel Muñoz, Huldrych F. Günthard, Niko Beerenwinkel, Amalio Telenti, Angela Ciuffi

Table of contents:

1. Modeling viral progression
2. Clustering of gene expression time courses
3. Supplementary Figures
4. Supplementary Tables

1. Modeling viral progression

The model. The raw measurements of viral intermediates consist of nine independent time series, each quantifying the progression of a specific step in the life cycle of the virus. Each representative marker (e.g., extracellular p24) accumulates in the sample from the start of infection until the time of the measurement. Ideally, each marker level, x_t, at time point t starts from zero and monotonically increases up to its maximum as more and more cells go through the corresponding phase of the viral life cycle. The net viral activity, $\bar{v}$, during time span Δt can be estimated as

(Eq. 5) $$\bar{v}_{t,t+\Delta t} = \frac{x_t - x_{t+\Delta t}}{\Delta t}$$

In practice, however, the measurements are not monotonic and linear estimation of **Eq. 5** can lead to negative viral activity estimates, which are biologically implausible given that the measured viral processes are not reversible. We address this problem by incorporating two further components into the model.

First, we account for a constant rate of marker loss during the experiment, which can be due to the experimental procedure or due to natural degradation of the marker in the sample. Marker loss is modeled by an exponential decay term, such that

(Eq. 6) $$\frac{x_t - x_{t+\Delta t}}{\Delta t} = \bar{v}_{t,t+\Delta t} - \lambda x_t, \quad \lambda \in [0,\infty)$$

where λ is the decay rate, and for $\lambda = 0$, **Eq. 5** is recovered. Second, we constrain the viral activity, v_t, to be a non-negative parametric function over time. Specifically, assuming that one

unit of marker (e.g., one 2LTR circle) is produced only after a sequence of sub-events has taken place, the distribution of the waiting time until production of one unit of the marker can be described by a gamma distribution function,

(Eq. 7) $$\Gamma(t;k,\theta) = \frac{1}{\theta^k \int_0^\infty u^{k-1} e^{-u} du} t^{k-1} e^{-t/\theta}, \quad k,\theta \in (0,\infty)$$

with shape and scale parameters k and θ, respectively. The overall expected production rate of the marker in the sample, v_t, is then proportional to the density function at time t. Using an additional normalization constant α this relationship can be written as

(Eq. 8) $$v_t^{(\theta,k)} = \alpha\Gamma(t;k,\theta) = \alpha\hat{v}_t^{(\theta,k)}, \quad \alpha \in [1,\infty)$$

where we have defined $\hat{v}_t = \hat{v}_t^{(\theta,k)} = \Gamma(t;k,\theta)$, and α corresponds to the number of identical sub-processes. For $\Delta t \to 0$, **Eq. 6** becomes the following ordinary differential equation (ODE)

(Eq. 9) $$\frac{dx}{dt} = v_t^{(\theta,k)} - \lambda x_t$$

Assuming $x_0 \geq 0$, this deterministic equation can be solved as

(Eq. 10) $$x_t = e^{-\lambda t}\left[\int_0^t v_{t'}^{(\theta,k)} e^{\lambda t'} dt' + x_0\right] \quad x_0 \in [0,\infty)$$

The normalization constant, α, can be factored out of the equation,

(Eq. 11) $$x_t = \alpha e^{-\lambda t}\left[\int_0^t \hat{v}_{t'}^{(\theta,k)} e^{\lambda t'} dt' + \hat{x}_0\right] \quad \hat{x}_0 = \frac{x_0}{\alpha}$$

We denote the right hand side of this equation by $f\left(\hat{v}_t^{(\theta,k)}, \lambda, \hat{x}_0, \alpha\right)$. **Eq. 11** allows for deriving the pattern of viral activity in the absence of an absolute measure of the marker, which is often the case, for instance, when the data is produced by qPCR relative to an internal reference substrate. The viral activity model consists of four main components, namely activity shape ($\hat{v}_t^{(\theta,k)}$), marker decay (λ), initial marker ($\hat{x}_0$), and scaling constant (α). In the absence of marker decay, the model reduces to a linear transformation of the cumulative gamma distribution function.

Parameter estimation and confidence intervals. For each of the viral activity markers, model parameters were estimated using a least squares fit to the experimental data vector $\boldsymbol{y}$ with

(Eq. 12) $$\tau(y_t) = \tau\left(f\left(\hat{v}_t^{(\theta,k)}, \lambda, \hat{x}_0, \alpha\right)\right) + \varepsilon_t$$

where the function $\tau(.)$ is variance stabilizing transformation (described below), and $\boldsymbol{\varepsilon}$ is a vector of i.i.d. Gaussian noise. Confidence intervals of the peak viral activity, which is given by $k\theta$, were specifically computed by using parametric bootstrapping [1]. For this, we generated bootstrap samples from the model using the estimated Gaussian noise, and estimated the distribution of the peak viral activity directly from the bootstrap samples.

Variance stabilization. Viral data was produced on three main platforms, namely qPCR, FACS, and ELISA (**Figure S2)**. The data are heteroscedastic, which can significantly degrade the quality of the model fit. In order to address this problem, the logarithm was applied as a variance stabilizing transformation prior to least squares fitting. This choice is well-justified in case of qPCR data, which comprises seven out of the nine data sets. As **Figure S3** illustrates, the variance in the qPCR values can be almost perfectly described by the mean using a set of parallel regression lines on a logarithmic scale with data set-specific intercepts, b_j, and a common slope, a, corresponding to the equation

$$\text{(Eq. 13)} \qquad \mathrm{var}\left(y_{j,m}\right) = e^{b_i} \bar{y}_{j,m}^{a}$$

where $\bar{y}_{j,m}$ is the mean value of the technical replicates of the mth measurement in the jth set of qPCR measurements. Hence, the variance stabilizing transformation, τ, can be found as

$$\text{(Eq. 14)} \qquad \tau\left(y_j\right) = e^{-\frac{b_j}{2}} \int^{y} \omega^{-\frac{a}{2}} d\omega$$

The constant exponential term can be disregarded in practice, and the integral in **Eq. 14** becomes the log transformation for a slope parameter value of $a = 2$. Log transformation of qPCR data is identical to using the raw −ΔΔCT values before the exponentiation step that is usually employed prior to reporting experimental values. For the FACS and ELISA datasets, there was not enough data available for *de novo* derivation of a suitable transformation. Hence, the log transformation was chosen in accordance with common practice [2,3].

Model fitting and further remarks. In order to fit the model to the experimental data, we use the trust region algorithm for nonlinear least-squares optimization implemented in Matlab [4]. At each iteration of the algorithm, the value of x_t was calculated numerically from the ODE in **Eq.9** using the Dormand-Prince method for solving non-stiff ODEs [5] implemented in the Matlab *ode45* function. All parameters were searched over the domain $[0, +\infty)$, which spans the valid domain of the function parameters. We used a difference of 10^{-10} in subsequently updated parameters as a threshold to detect convergence of the algorithm. Each set of measurements was once used with the full model, and once with a reduced model in which the decay constant was fixed to zero. The likelihood was then calculated for the full and the reduced model and the preferred model was chosen based on the Bayesian Information Criterion (BIC) score [6]. **Figure S4** illustrates the model fits to data. **Figure S5** depicts the extracted pattern of activity and progression for each of the viral life steps. Bootstrapping was performed over the whole procedure using 500 samples. The resulting bootstrap distribution is shown by the white inner violins for the peak activity of each viral step in **Figure 1B**. We repeated the modeling

using three alternative activity shapes namely, Weibull, lognormal, and truncated normal distribution functions, all of which yielded very similar results (results not shown here). In addition to the conventional confidence intervals based on the Jacobian matrix, and the parametric bootstrapping, we further assessed predictive power and stability of the fits qualitatively by fitting the model to data after removing the last time points. **Figure S6** illustrates the resulting fits for the case of the last, and two last time points removed.

2. Clustering of gene expression time courses

Gene expression profiles over the 24h observation time were clustered to identify co-regulated sets. For this purpose, we analyzed all 7,991 genes that were significantly described by the regression model, i.e., for which at least one regression coefficient in w_i was significantly different from zero, defined by a q-value below 0.05. Each gene *i* was represented by a normalized vector of regression weights, $\hat{w}_i$, such that

(Eq. 16) $$\hat{w}_i = \frac{w_i}{\|w_i\|}$$

We used the squared Euclidian distance for measuring the similarity of gene expression profiles. This choice is equivalent to the cosine distance between two un-normalized data vectors. This distance measure is scale-independent and sensitive to sign changes (additive constants). By contrast, the correlation distance would be insensitive to downregulation and upregulation as long as the relative expression pattern is unchanged. Clustering was carried out using the k-means algorithm [7] repeated 10,000 times using different sets of randomly chosen cluster seeds, and the best clustering was selected based on the lowest sum of point-to-cluster distances.

The optimal number of clusters was chosen based on the BIC score [6] calculated as

(Eq. 17) $$BIC(k) = -2\ln(L_k) + p_k \ln(N)$$

for N observations, the data likelihood, L_k, evaluated at the maximum likelihood parameters, p_k degrees of freedom, and k clusters. The likelihood was derived under the assumption of identical spherical Gaussian distributions of the length-normalized weight vectors [8]. The number of free parameters, p_k, was counted as the sum of $(k - 1)$ cluster probabilities, $3k$ centroid coordinates, and one intra-cluster variance parameter [8]. Calculating the BIC score over a range of k, one can select an optimal number of clusters, k^*, as

(Eq. 18) $$k^* = \underset{k}{\arg\min}\ BIC(k)$$

However, in order to account for the uncertainty in the data and the non-deterministic nature of the k-means algorithm, we add a bootstrapping scheme for choosing the optimal number of clusters. For each tested parameter value, k, bootstrap datasets were produced using re-sampling. Clustering was performed, and the corresponding BIC score was calculated for each bootstrap dataset separately. Sample average, B_k, and standard deviation, δ_k, of the bootstrap BIC scores

were calculated for each tested value of k. Accounting for additional uncertainty in the mean estimator, we defined the following quantity:

(Eq. 19) $$s_k = \delta_k \sqrt{1 + \frac{1}{\beta}}$$

with β denoting the number of bootstrap samples. The number of clusters, k^+, was chosen as the most parsimonious solution within one standard deviation from the global minimum average BIC,

(Eq. 20) $$k^+ = \min \left\{ k \,\middle|\, B_k \le B_{k^*} + s_{k^*} \quad , \; k^* = \underset{k'}{\arg\min} \; B_{k'} \right\}$$

This 1-standard-error distance is a commonly used threshold [9]. We tested the quality of clustering for a range of k values between 1 and 50 and the number of clusters was chosen as $k^+ = 18$ (**Figure S7**). The resulting clusters are shown in **Figure S8**.

References

1. Efron B, Tibshirani RJ (1994) An Introduction to the Bootstrap.
2. Herzenberg LA, Tung J, Moore WA, Herzenberg LA, Parks DR (2006) Interpreting flow cytometry data: a guide for the perplexed. Nat Immunol 7: 681-685.
3. Peterman JH, Butler JE (1989) Application of theoretical considerations to the analysis of ELISA data. Biotechniques 7: 608-615.
4. Coleman TF, Li Y (1996) An Interior, Trust Region Approach for Nonlinear Minimization Subject to Bounds. SIAM Journal on Optimization 6: 418-445.
5. Dormand JR, Prince PJ (1980) A family of embedded Runge-Kutta formulae. J Comp Appl Math 6: 19-26.
6. Schwarz G (1978) Estimating the dimension of a model. Ann Satist 6: 461-464.
7. Jain AK, Dubes RC (1981) Algorithms for Clustering Data: Prentice-Hall.
8. Pelleg D, Moore A (2000) X-means: Extending K-means with Efficient Estimation of the Number of Clusters. International Conference on Machine Learning. Stanford, CA.
9. Tibshirani R, Walther G, Hastie T (2001) Estimating the number of clusters in a data set via the gap statistic. J R Stat Soc Ser B Stat Meth 63: 411-423.

3. Supporting Figure Legends

Figure S1. Experimental design.

Figure S2. Primary measurement data on nine viral intermediates. Progression was measured by qPCR (Early RT, Late RT, 2LTR, and Integrated viral DNAs), RT–qPCR (Multiply spliced, Singly spliced, and Unspliced viral transcripts), FACS (GFP mean fluorescent intensity), and ELISA (viral p24 release) every two hours and normalized with respect to the last time point (24hr).

Figure S3. Technical variance as a function of mean for qPCR data. The R^2 statistic represents the described variance by the seven parallel fitted lines sharing the same slope ($a = 1.99$), and having independent intercepts for each of the seven qPCR measurement sets.

Figure S4. Viral progression model fitted curves: Panel A illustrates the model fits in the variance stabilized coordinates (logarithmic scale). Panel B illustrated the fits when transformed back to the original coordinates of measurements (See figure S3). Blue dots indicate the experimental measurement values, and solid red lines are the fitted curves with the dashed lines representing the 95% confidence intervals of the fit derived by linear approximation around the fitted parameters using the Jacobian matrix.

Figure S5. Estimated activity and progression of viral replication steps. Accounting for the initial viral input, experimental noise, and decay of the measured species yields a refined estimation of activity rates (panel A) and the progression (panel B) of the viral cycle steps. The vertical dashed lines indicate the peak of activity. The values are normalized such that asymptotically progression reaches 100%.

Figure S6. Qualitative assessment of the predictive power and stability of the fits. Shown are the fits using full data (solid dark blue), data missing 24hr. (dashed blue), and data missing 22hr. and 24hr. (dashed light blue) time points extrapolated 10 hours beyond the original experiment. This mimics the case that the experiment was finished earlier than 24 hours. Fits derived after removing the last time point are all very close to the full model. This still remain the case for all viral steps after removing the two last time points, except for the case of viral release.

Figure S7. Determining the number clusters. BIC score (Mean ± one standard deviation calculated from 500 bootstrapped samples) of the clustering for different numbers of clusters (k). The global minimum of the BIC was reached at $k^* = 23$ (red asterisk). The number of clusters, $k^+ = 18$ (green asterisk), was chosen as the most parsimonious solution within one standard deviation from the global minimum (dashed red line).

Figure S8. Clusters of host genes correlated with viral progression. Temporal expression patterns of 7991 genes were grouped into 18 clusters with differential expression profiles at three phases of the viral life cycle, namely reverse transcription, integration, and late phase. The boxplots on the left show the distribution of the normalized regression weights for each viral phase used in clustering. Cluster codes were defined for the three phases using characters ‘+’ and ‘−’ marking significant ($p < 10^{-2}$) upregulation and downregulation, respectively, and ‘o’

indicating no significant deviation. In total, six upregulated clusters (A), four clusters with mixed patterns of regulation (B), and eight downregulated clusters (C) were found. Expression change pattern of each gene over time is plotted for mock and HIV-1 samples (dotted orange lines) along with the cluster median (bold red line). Details of clusters are available at the dedicated web resource.

Figure S9. Expected population-level changes due to viral integration. Shown is the empirical null distribution of expression in mock samples and the corresponding empirical p-value boundaries. The blue line represents the expected population effect of viral integration assuming expression knock-out in the host gene (first scenario). The red line represents the expected population effect of viral integration assuming an increase in expression in the host gene by a factor of 105 (second scenario). Given the expected frequency of viral integrations, neither of these two opposite extreme scenarios implies significant perturbation in the population-level expression as compared to the variation in expression of the mock samples.

Figure S10. Infection efficiency assessed by FACS analysis of GFP expression. SupT1 cells (red and orange lines) or activated primary CD4+ T cells (dark and light blue lines) were transduced by VSV-G pseudotyped HIV vector carrying a *GFP* reporter gene. SupT1 cell infection was carried out with 3 μg p24 equivalent of HIV-based vector, either competent (red line), or heat-inactivated (hi; red dash line) or a 1:10 mix of competent and heat-inactivated virus (orange line). Expression of GFP was assessed by FACS over time. Data from original infection of SupT1 cells were also plotted (black line).

Figure S11. Correlation analysis between SAGE-Seq and RT-qPCR. Fourteen genes representative of diverse clusters were compared between the two techniques used, SAGE-Seq and RT-qPCR, by plotting the $\log_2$ fold change of HIV-1 over mock. Each dot corresponds to one selected gene at one time point. Correlation analysis of the $\log_2$ fold change of HIV-1 over mock was calculated by linear regression as $r^2 = 0.5183$, $p < 10^{-4}$. (Spearman $r = 0.7869$, $p < 10^{-4}$). Correlation improves towards late time points from $r^2 = 0.23$ at 2 hours ($p = 0.01$) to $r^2 = 0.77$ at 24 hours ($p < 10^{-4}$).

Figure S1

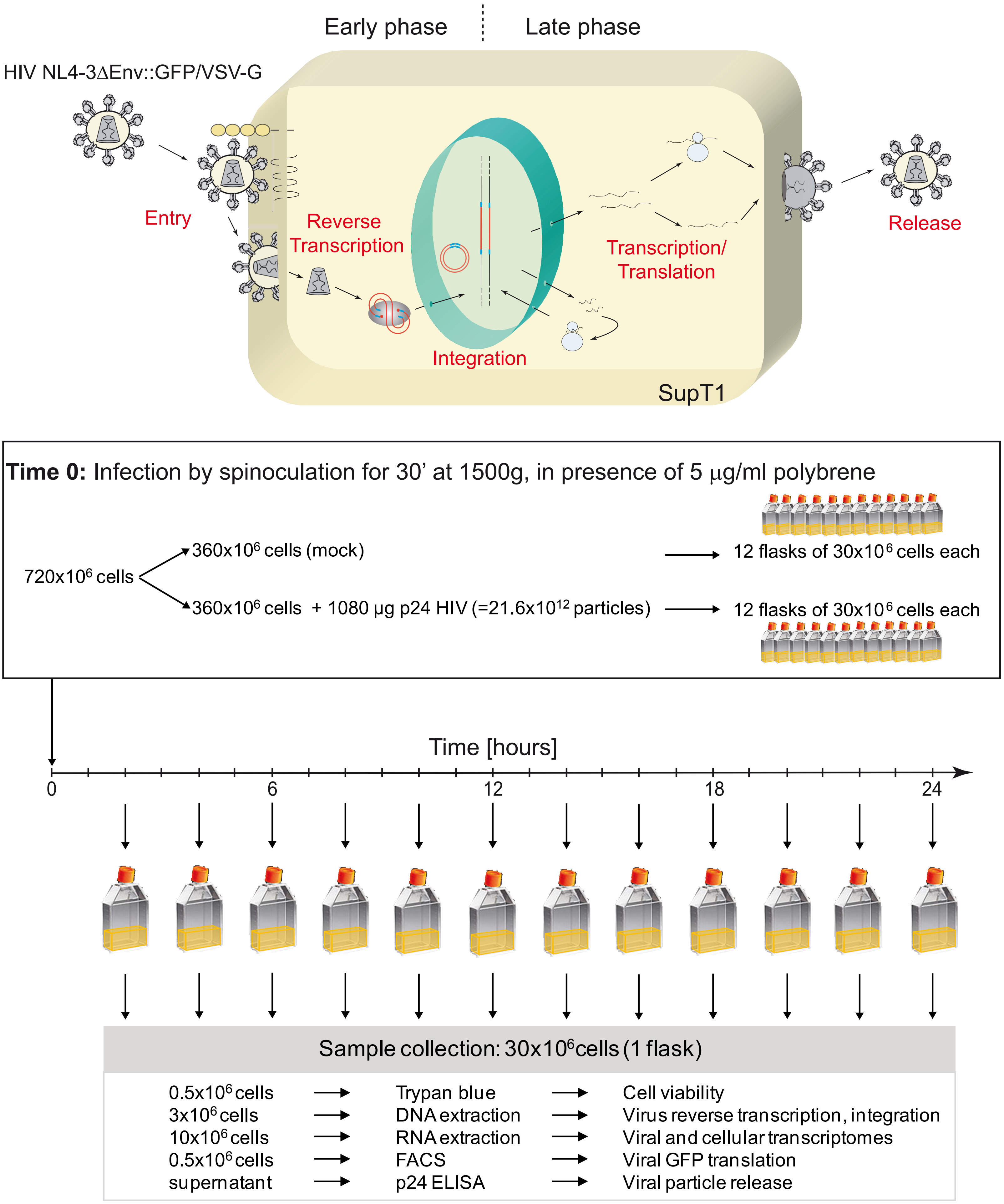
Early phase
Late phase
HIV NL4-3ΔEnv::GFP/VSV-G
Entry
Reverse Transcription
Integration
Transcription/ Translation
Release
SupT1
Time 0: Infection by spinoculation for 30' at 1500g, in presence of 5 μg/ml polybrene
720x10^6 cells
360x10^6 cells (mock)
360x10^6 cells + 1080 μg p24 HIV (=21.6x10^12 particles)
12 flasks of 30x10^6 cells each
12 flasks of 30x10^6 cells each
Time [hours]
0
6
12
18
24
Sample collection: 30x10^6cells (1 flask)
0.5x10^6 cells → Trypan blue → Cell viability
3x10^6 cells → DNA extraction → Virus reverse transcription, integration
10x10^6 cells → RNA extraction → Viral and cellular transcriptomes
0.5x10^6 cells → FACS → Viral GFP translation
supernatant → p24 ELISA → Viral particle release

Figure S2

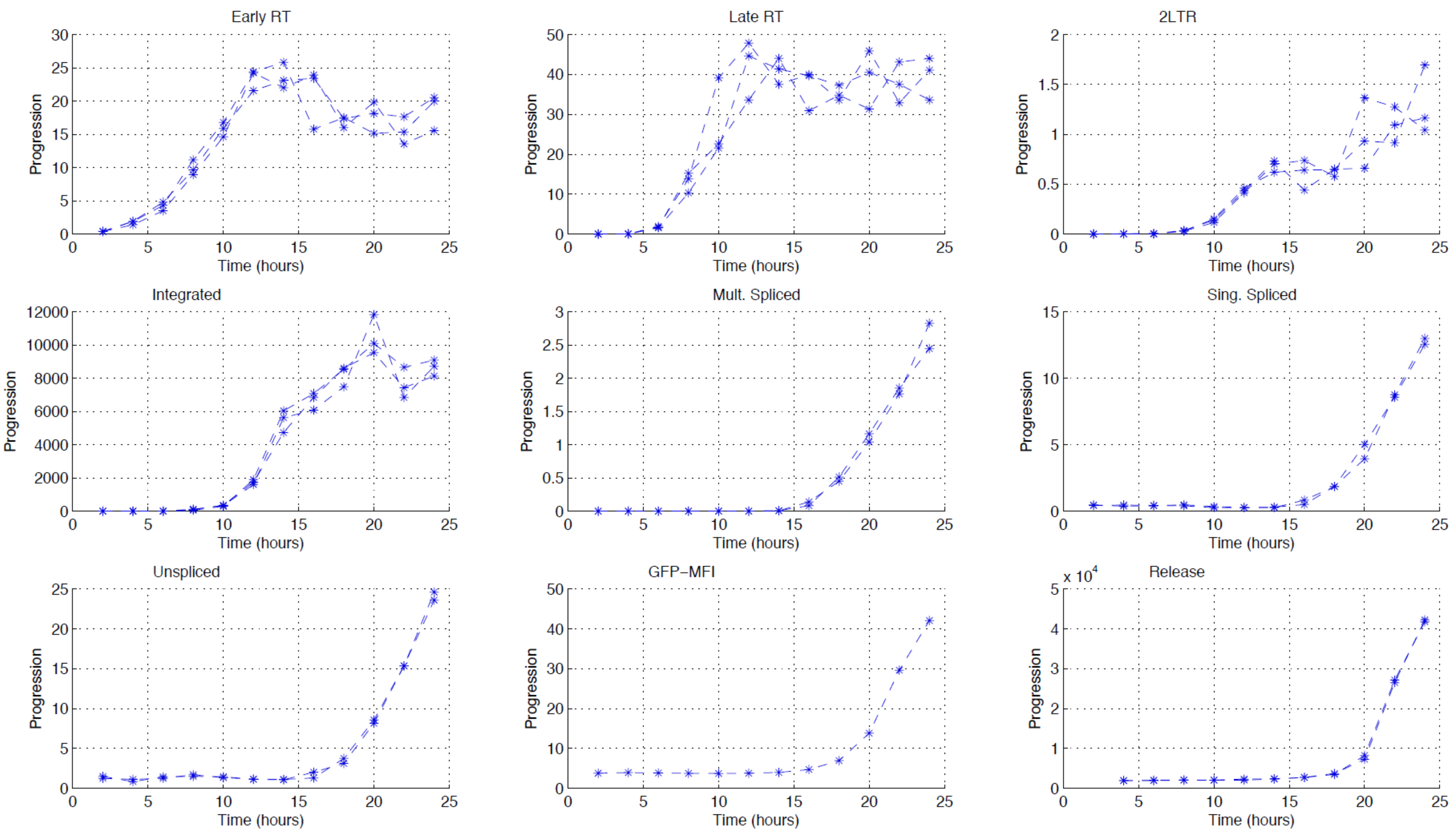
Early RT
Late RT
2LTR
Integrated
Mult. Spliced
Sing. Spliced
Unspliced
GFP−MFI
Release
x 10^4
Progression
Time (hours)

Figure S3

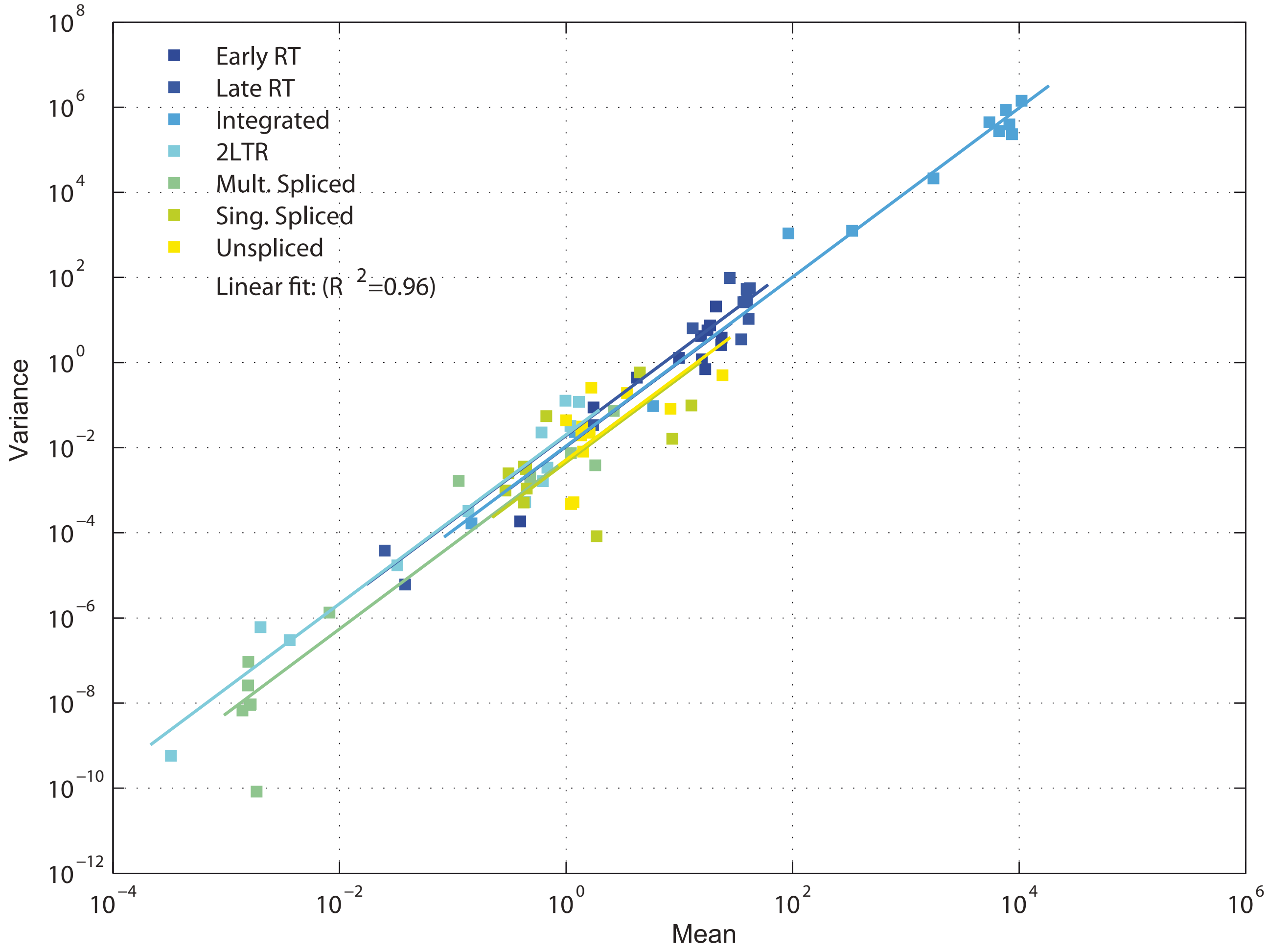

Early RT
Late RT
Integrated
2LTR
Mult. Spliced
Sing. Spliced
Unspliced
Linear fit: (R^2=0.96)
Variance
Mean
10^{8}
10^{6}
10^{4}
10^{2}
10^{0}
10^{-2}
10^{-4}
10^{-6}
10^{-8}
10^{-10}
10^{-12}
10^{-4}
10^{-2}
10^{0}
10^{2}
10^{4}
10^{6}

Figure S4

A

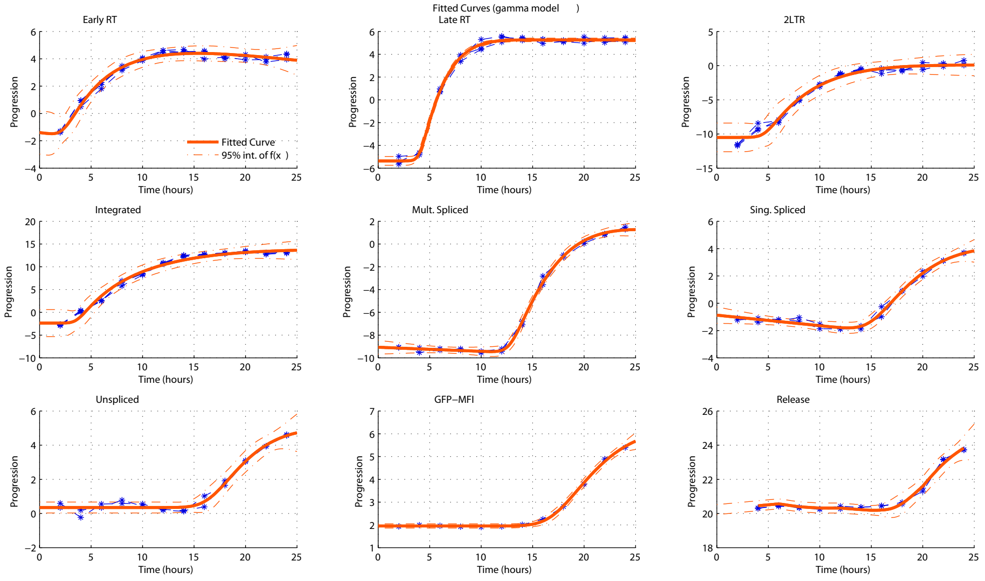
Fitted Curves (gamma model)
Early RT
Late RT
2LTR
Integrated
Mult. Spliced
Sing. Spliced
Unspliced
GFP−MFI
Release
Fitted Curve
95% int. of f(x)
Progression
Time (hours)

B

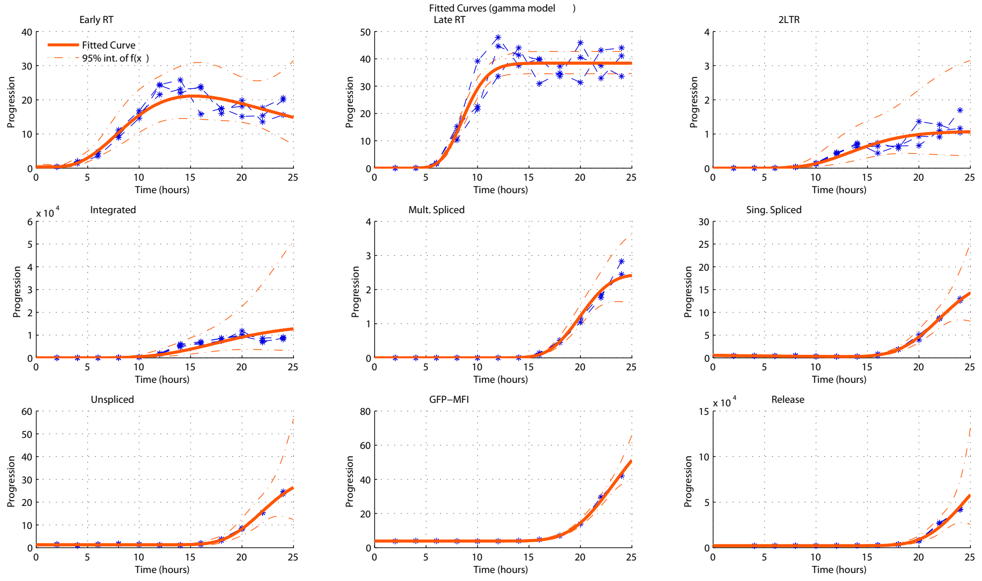
Fitted Curves (gamma model)
Early RT
Late RT
2LTR
Integrated
Mult. Spliced
Sing. Spliced
Unspliced
GFP−MFI
Release
Fitted Curve
95% int. of f(x)
Progression
Time (hours)

Figure S5

A

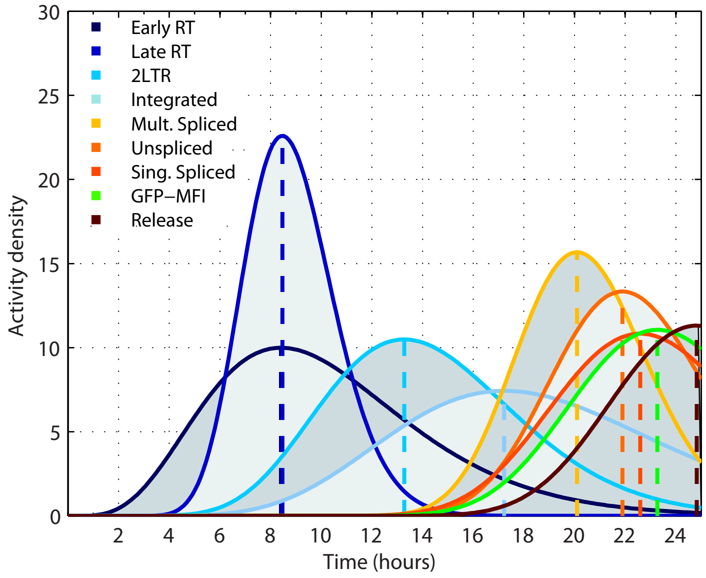
Early RT
Late RT
2LTR
Integrated
Mult. Spliced
Unspliced
Sing. Spliced
GFP–MFI
Release
Activity density
Time (hours)
30
25
20
15
10
5
0
2
4
6
8
10
12
14
16
18
20
22
24

B

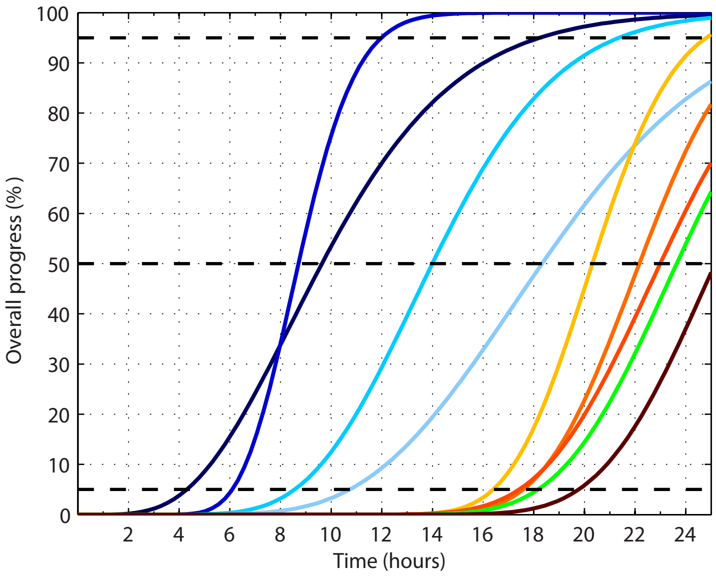
Overall progress (%)
Time (hours)
100
90
80
70
60
50
40
30
20
10
0
2
4
6
8
10
12
14
16
18
20
22
24

Figure S6

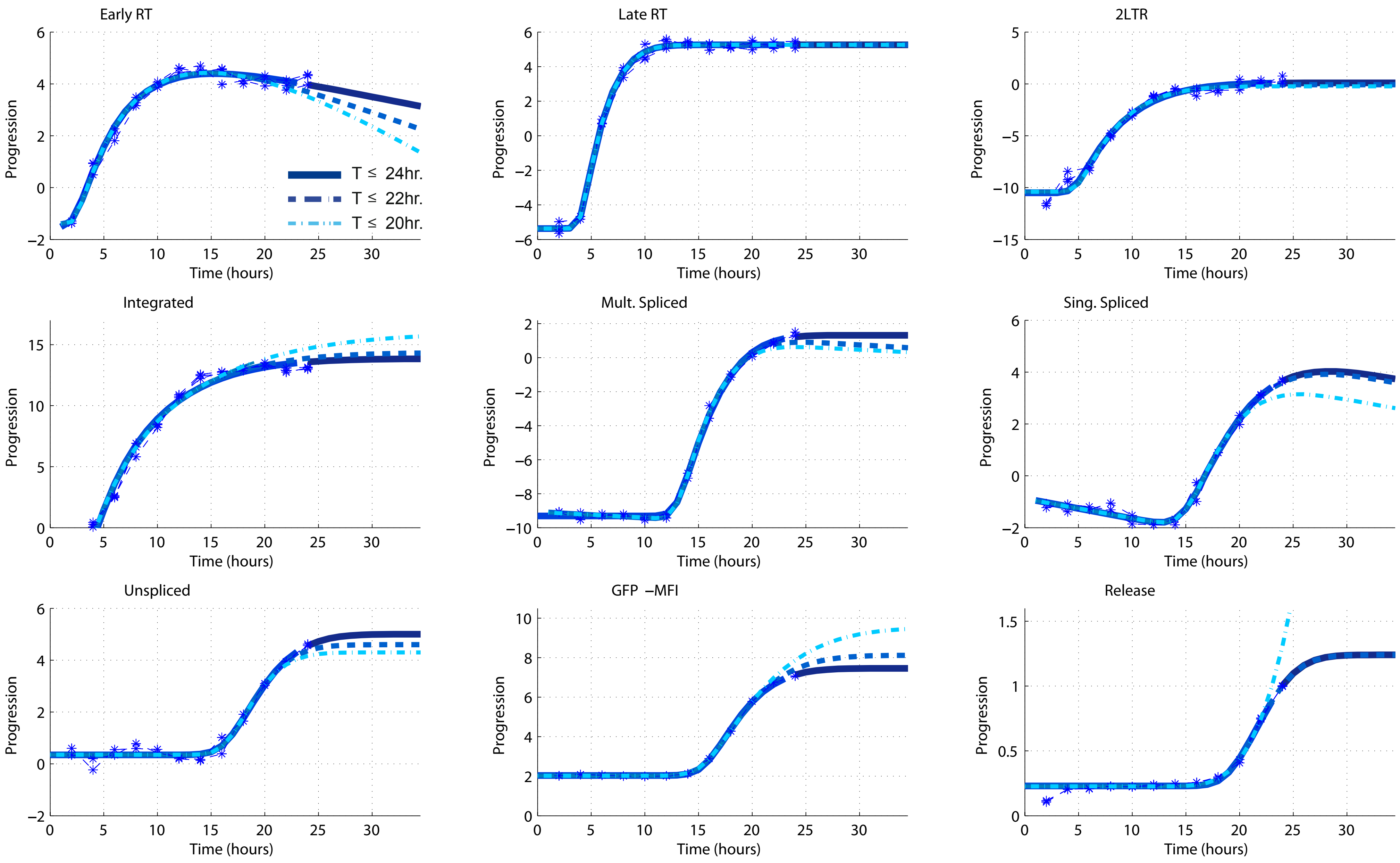
Early RT
Late RT
2LTR
Integrated
Mult. Spliced
Sing. Spliced
Unspliced
GFP −MFI
Release
Progression
Time (hours)
T ≤ 24hr.
T ≤ 22hr.
T ≤ 20hr.

Figure S7

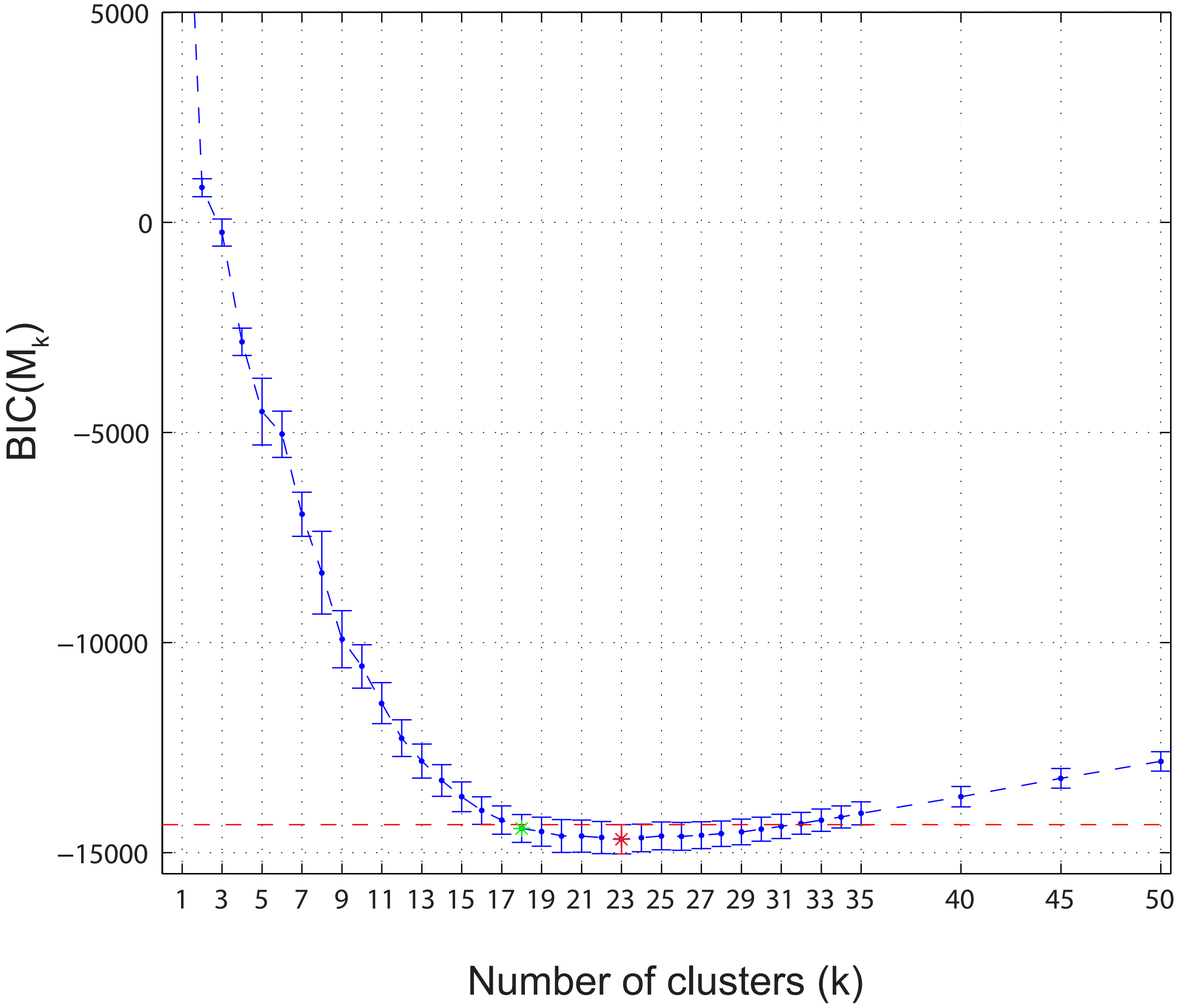

5000
0
−5000
−10000
−15000
BIC(M$_k$)
1 3 5 7 9 11 13 15 17 19 21 23 25 27 29 31 33 35 40 45 50
Number of clusters (k)

Figure S8

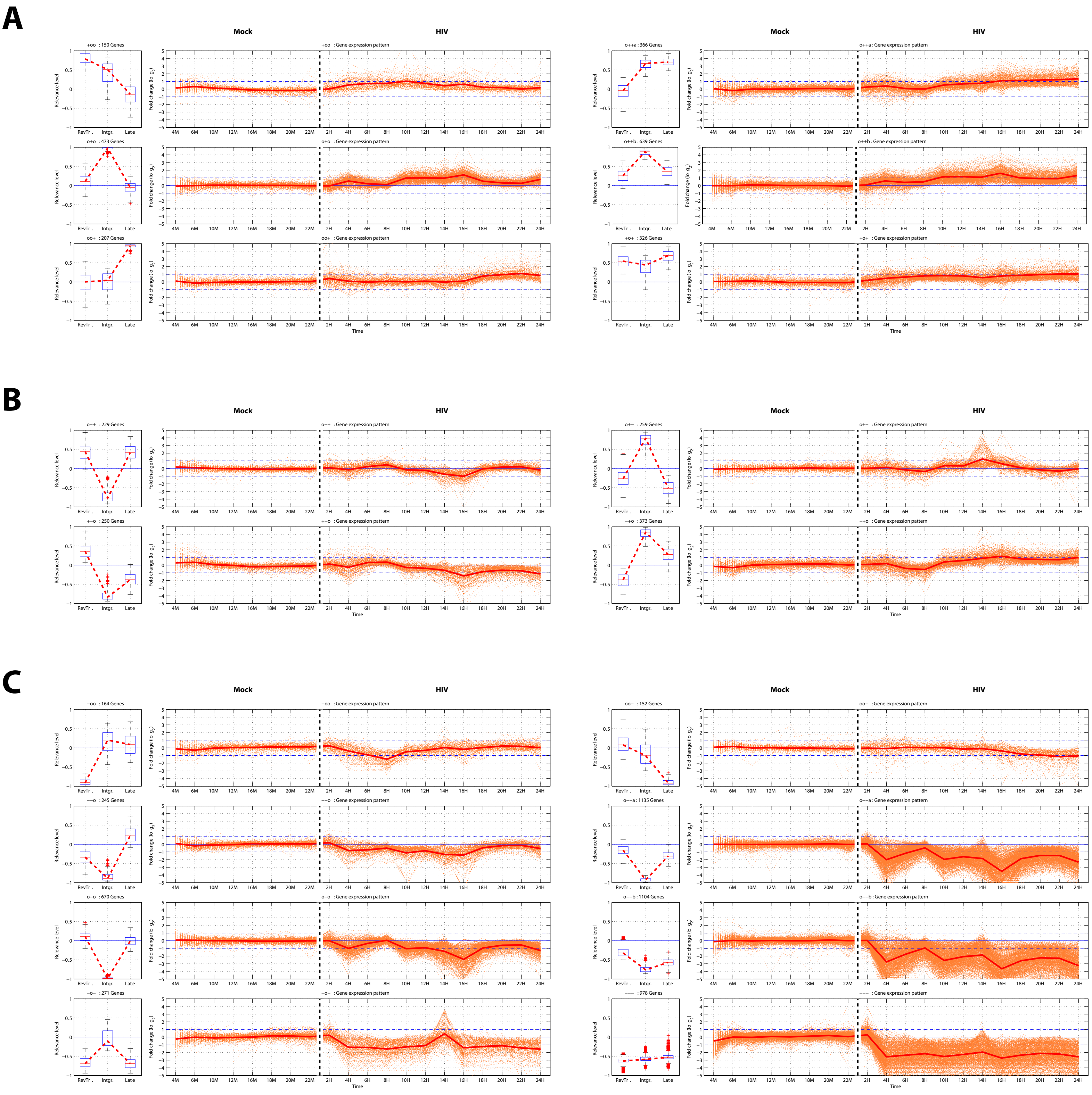

Figure S9

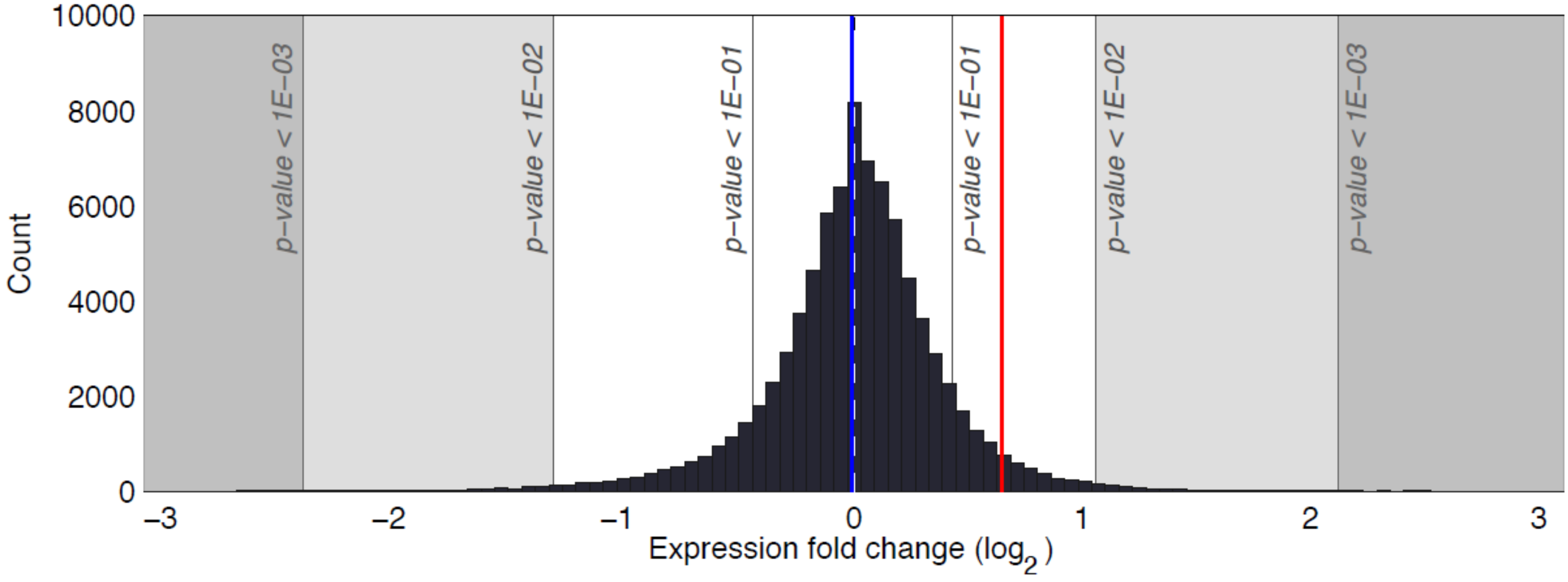

10000
8000
6000
4000
2000
0
Count
p−value < 1E−03
p−value < 1E−02
p−value < 1E−01
p−value < 1E−01
p−value < 1E−02
p−value < 1E−03
−3
−2
−1
0
1
2
3
Expression fold change (log2)

Figure S10

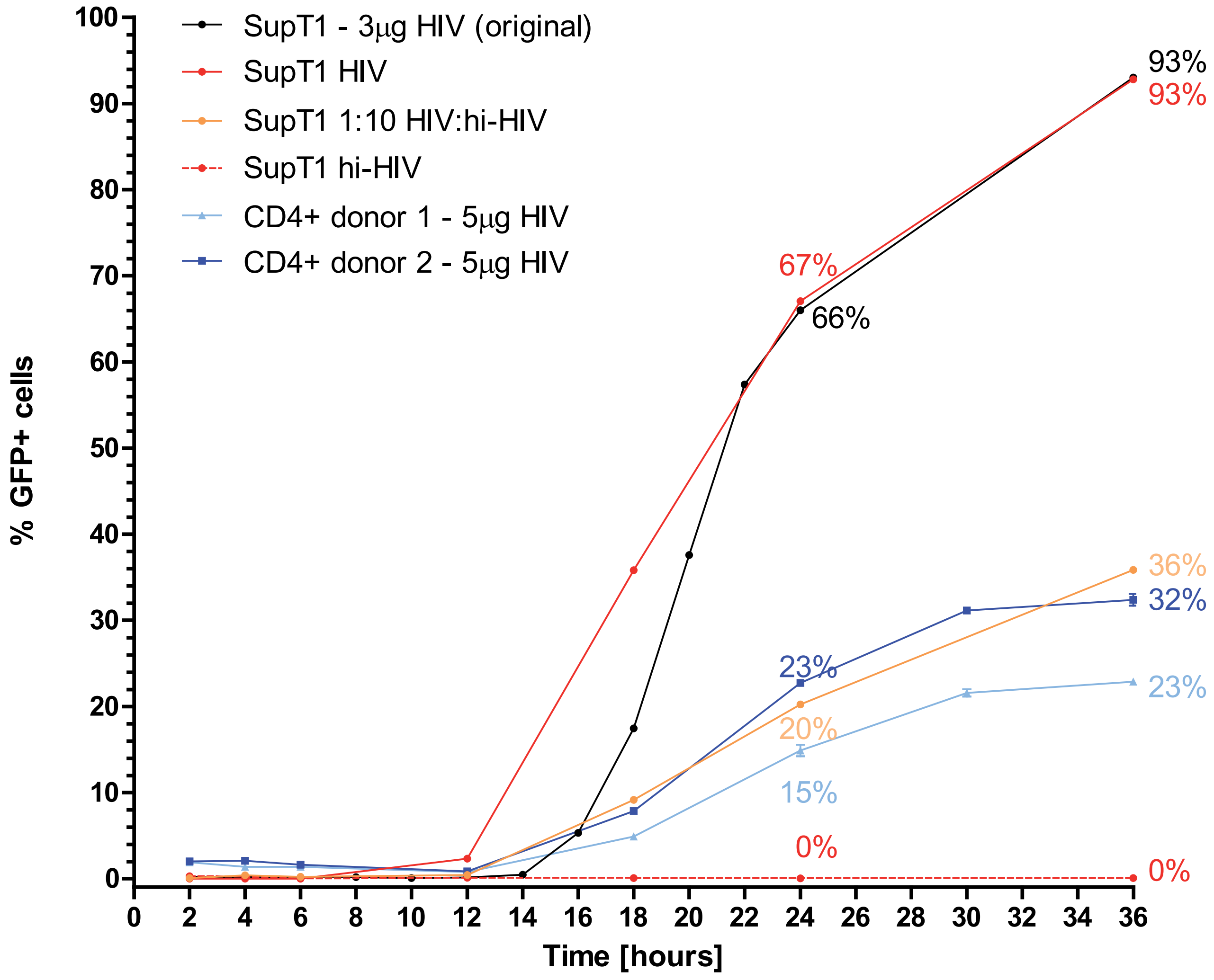

SupT1 - 3μg HIV (original)
SupT1 HIV
SupT1 1:10 HIV:hi-HIV
SupT1 hi-HIV
CD4+ donor 1 - 5μg HIV
CD4+ donor 2 - 5μg HIV
% GFP+ cells
Time [hours]
0
10
20
30
40
50
60
70
80
90
100
0
2
4
6
8
10
12
14
16
18
20
22
24
26
28
30
32
34
36
93%
93%
67%
66%
36%
32%
23%
23%
20%
15%
0%
0%

Figure S11

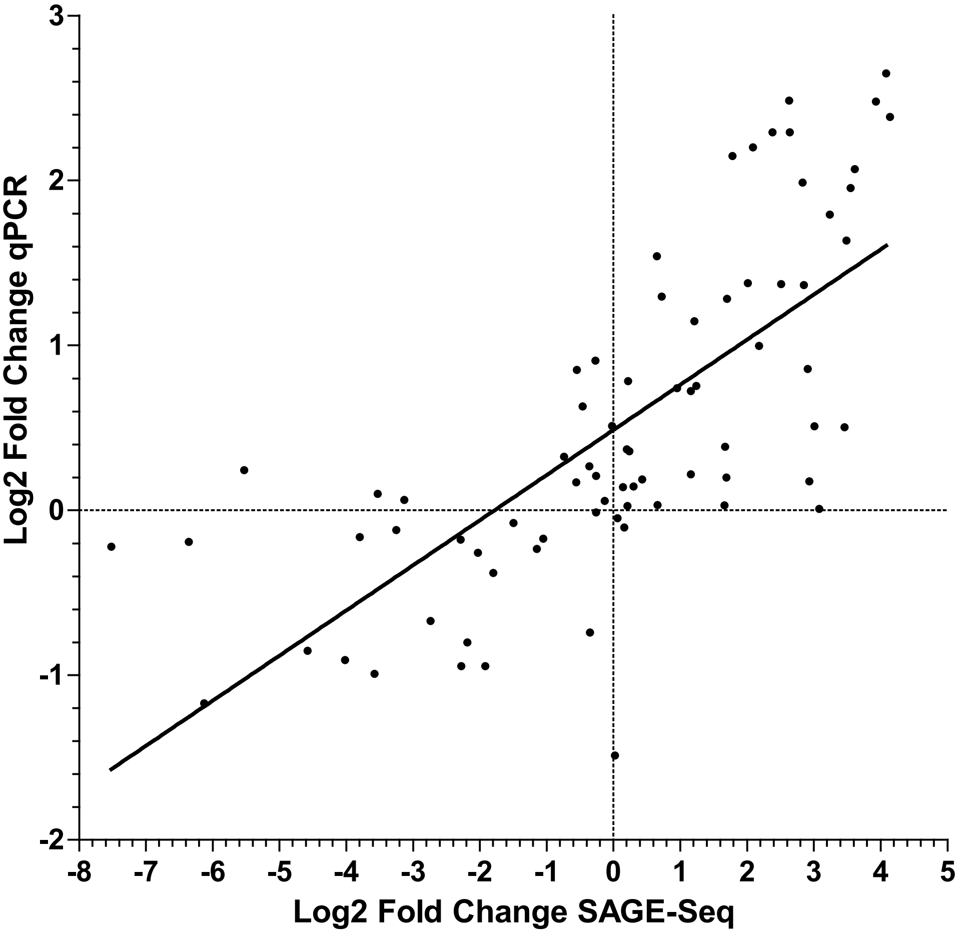
Log2 Fold Change qPCR
3
2
1
0
-1
-2
-8
-7
-6
-5
-4
-3
-2
-1
0
1
2
3
4
5
Log2 Fold Change SAGE-Seq

4. Supplementary Tables

Supplementary Tables S1 and S2

Analysis of transcriptional regulators. Of 1391 transcription factors from the curated set defined by Vaquerizas et al. [1]**,** 612 were found expressed, of which 421 (69%) were modulated in concordance with viral progression features, with 38%, 21%, and 10% in down-, up-, and mixed regulated clusters, respectively; a pattern shared with the general distribution of cellular transcripts. Genes sharing transcription factor binding motifs were inspected for evidence of co-regulation. Nine gene clusters were found to be enriched in targets of at least one transcription factor. Out of the total 25 cognate transcription factors identified with co-regulated target genes, 17 were expressed in our system and 10 of them showed the same direction of modulation in expression as expected from their target set (**Table S1**). We detected 399 cellular miRNAs from the miRbase database [2], out of which 176 (44%) showed modulation through viral progression. The distribution across clusters of expressed miRNAs was 17%, 17%, and 10% in down-, up-, and mixed-regulated clusters, respectively, indicating a lower enrichment of miRNAs in down-regulated clusters compared to the distribution of cellular mRNAs ($p < 10^{-7}$). Genes sharing 3'UTR miRNA binding sites were inspected for evidence of co-regulation. Seven gene clusters were found to be enriched for a number of miRNA targets. Out of the total 17 miRNA motifs identified with co-regulated target genes, 14 were expressed in our system. Four of them (let7b, miR101, miR124, miR142) showed the expected modulation in expression that could be expected from the predicted target set (**Table S2**).

References

1. Vaquerizas JM, Kummerfeld SK, Teichmann SA, Luscombe NM (2009) A census of human transcription factors: function, expression and evolution. Nat Rev Genet 10: 252-263.
2. Kozomara A, Griffiths-Jones S (2011) miRBase: integrating microRNA annotation and deep-sequencing data. Nucleic Acids Res 39: D152-157.

Table S1 : Enriched sets of transcription factor target genes

TF target set	Enriched cluster pattern	Expressed TF	TF pattern
V$PPAR_DR1_Q2	- - -	PPARA	o++a
V$E2F_03	o--a	ND	
V$TEL2_Q6	o--a	ETV7	
V$NRF2_01	o--a	GABPB1	+-o
RCGCANGCGY_V$NRF1_Q6	o--a	NRF1	NA
GGGCGGR_V$SP1_Q6	o--a	SP1	o--a
V$E2F_Q6_01	o--a	TFDP1/E2F	-o-
V$NFMUE1_Q6	o--b	ND	
V$E2F1_Q6_01	o--b	E2F1	o--b
V$E2F1_Q4	o--b	E2F1	o--b
TGTTTGY_V$HNF3_Q6	o--b	FOXA1	
GGGAGGRR_V$MAZ_Q6	o--b	MAZ	o--a
V$MYCMAX_01	o--b	MYC	o--b
CGTSACG_V$PAX3_B	o--b	PAX3	
V$E2F_Q3_01	o--b	TFDP1/E2F	-o-
V$E2F1_Q4_01	o--b	TFDP1/E2F	-o-
V$ATF6_01	oo-	ATF6	o--a
V$CDX2_Q5	oo-	CDX2	
V$CREB_Q4	oo-	CREB1	o+-
V$CREB_Q2_01	oo-	CREB1	o+-
TGGAAA_V$NFAT_Q4_01	oo-	NFAT	o--a, o--b
V$OCT1_06	oo-	POU2F1	NA
V$YY1_01	oo-	YY1	o--b
CGTSACG_V$PAX3_B	-oo	PAX3	
V$SRF_Q6	-oo	SRF	o+-
V$RORA1_01	o+-	RORA	NA
V$YY1_01	o+-	YY1	o--b
V$USF_C	+-o	ND	
V$CEBP_01	+-o	CEBPA	
V$CEBPB_01	+-o	CEBPB	+-o
V$GATA6_01	+-o	GATA6	
V$OCT1_07	-+o	POU2F1	NA
V$CREBP1_01	+oo	ATF2	NA
V$HLF_01	+oo	HLF	
V$E4BP4_01	+oo	NFIL3	-+o
TTAYRTAA_V$E4BP4_01	+oo	NFIL3	-+o

ND: not detected

NA: not associated with viral progression features

Table S2 : Enriched sets of miRNA target genes

Enriched sets in clusters	Enriched Cluster pattern	Expressed cognate miRNAs	miRNA pattern
Targets of let-7b-5p	o--a	hsa-let-7b	+o+
Targets of MIR 124-3p	o--a	hsa-miR-124	+oo
Targets of MIR 7-5p	o--a	hsa-miR-7	-o-
Targets of MIR 133a	o--b	ND	
Targets of MIR 16-5p	o--b	hsa-miR-16	NA
Targets of MIR 34a-5p	o--b	ND	
Targets of MIR 98	o--b	hsa-miR-98	NA
Targets of MIR 99a-5p	o--b	hsa-miR-99a	NA
Targets of MIR 30	--o	hsa-miR-30e, hsa-miR-30b	oo-
Targets of MIR 130b-3p	oo-	hsa-miR-130b	oo-
Targets of MIR 19b-3p	oo-	hsa-miR-19b	oo-
Targets of MIR 335-5p	-+o	ND	
Targets of MIR 101-3p	o+-	hsa-miR-101	o--a
Targets of MIR 125a-5p	+oo	hsa-miR-125a-5p	+o+
Targets of MIR 192-5p	+o+	hsa-miR-192	+oo
Targets of MIR 21-5p	+o+	hsa-miR-21	NA
Targets of MIR 101-3p	o++b	hsa-miR-101	o--a
Targets of MIR 142-3p	o++b	hsa-miR-142-3p	oo-
Targets of MIR 192-5p	o++b	hsa-miR-192	+oo

ND: not detected

NA: not associated with viral progression features

Table S3 : Oligonucleotides used in the study				
**Primer name**	**5'-3' primer sequence**	**Orientation**	**Target (HIV position)**	**Purpose**
MA.pr-243	GTGCCCGTCTGTTGTGTGAC	fwd	U5 (560-579)	early RT
MA.pr-244	GGCGCCACTGCTAGAGATTT	rev	U5-PBS (642-623)	early RT
MA.pr-275	CTAGAGATCCCTCAGACCCTTTTAGTCAGTGTGG	FAM-TAMRA probe	U5 (588-621)	early RT
MA.pr-245	TGTGTGCCCGTCTGTTGTGT	fwd	U5 (557-576)	late RT
MA.pr-246	GAGTCCTGCGTCGAGAGATC	rev	psi (699-680)	late RT
MA.pr-276	CAGTGGCGCCCGAACAGGGA	FAM-TAMRA probe	PBS (633-652)	late RT
MA.pr-247	AACTAGGGAACCCACTGCTTAAG	fwd	R (9428-9450)	2-LTR circles
MA.pr-248	TCCACAGATCAAGGATATCTTGTC	rev	U3 (51-28)	2-LTR circles
MA.pr-276	ACACTACTTTGAGCACTCAAGGCAAGCTTT	FAM-TAMRA probe	R-U5 (9458-9487)	2-LTR circles
MA.pr-249	GCCTCCCAAAGTGCTGGGATTACA	fwd	Alu (host)	integrated, first PCR
MA.pr-250	GCTCTCGCACCCATCTCTCTCC	rev	gag (803-782)	integrated, first PCR
MA.pr-251	GCCTCAATAAAGCTTGCCTTGA	fwd	R (522-543)	integrated, nested PCR
MA.pr-252	TCCACACTGACTAAAAGGGTCTGA	rev	U5 (622-599)	integrated, nested PCR
MA.pr-294	CCCGTCTGTTGTGTGACTCTGGTAACTAG	FAM-TAMRA probe	U5 (563-591)	integrated, nested PCR
MA.pr-253	AAGGGATTCACTCAGGCTCTTTC	fwd	intron 5 (4308-4330)	HMBS (PBGD) (GeneID:3145)
MA.pr-254	GGCATGTTCAAGCTCCTTGG	rev	exon 5 (4382-4363)	HMBS (PBGD) (GeneID:3145)
MA.pr-279	CCGGCAGATTGGAGAGAAAAGCCTGT	VIC-MGB_NFQ probe	intron 5-exon 5 (4334-4359)	HMBS (PBGD) (GeneID:3145)
mf84-AK145	ACAGTCAGACTCATCAAGCTTCTCTATCAAAGCA	fwd	tat1/rev1 (6011-6044)	multiply spliced (1.8/2 kb class)
mf83	GGATCTGTCTCTGTCTCTCTCTCCACC	rev	env/rev2 (8302-8276)	multiply spliced (1.8/2 kb class)
mf226	AGGGGACCCGACAGGCCC	FAM-TAMRA probe	env/tat2/rev2 (8240-8257)	multiply spliced (1.8/2 kb class)
mf222	GGCAGGGATATTCACCATTATCGTTTCAGA	fwd	env (8193-8222)	singly spliced (4 kb class) + unspliced (9 kb class)
mf83	GGATCTGTCTCTGTCTCTCTCTCCACC	rev	env/rev2 (8302-8276)	singly spliced (4 kb class) + unspliced (9 kb class)
mf226	AGGGGACCCGACAGGCCC	FAM-TAMRA probe	env/tat2/rev2 (8240-8257)	singly spliced (4 kb class) + unspliced (9 kb class)
mf299	GCACTTTAAATTTTCCCATTAGTCCTA	fwd	pol (2536-2562)	unspliced (9 kb class)
mf302	CAAATTTCTACTAATGCTTTTATTTTTTC	rev	pol (2662-2634)	unspliced (9 kb class)
mf348	AAGCCAGGAATGGATGGCC	FAM-TAMRA probe	pol (2586-2604)	unspliced (9 kb class)
mf45	TCGACAGTCAGCCGCATCTT	fwd	exon 1 (45-64)	GAPDH (GeneID: 2597)
mf46	GGCAACAATATCCACTTTACCAG	rev	exon 3 (2070-2048)	GAPDH (GeneID: 2597)
mf70tq	AAGGTCGGAGTCAACGGATTTGGTCGT	FAM-TAMRA probe	exon 2-exon 3 (355-371/2004-2013)	GAPDH (GeneID: 2597)
MA.pr-524	[Phosp]TAGTCCCTTAAGCGGAG-[AmC7-Q]	sense		integration site determination, linker Mse
MA.pr-525	GTAATACGACTCACTATAGGGCTCCGCTTAAGGGAC	antisense		integration site determination, linker Mse
MA.pr-526	[Phosp]GTCCCTTAAGCGGAG-[AmC7-Q]	sense		integration site determination, linker Nla
MA.pr-527	GTAATACGACTCACTATAGGGCTCCGCTTAAGGGACCATG	antisense		integration site determination, linker Nla
MA.pr-528	CTTAAGCCTCAATAAAGCTTGCCTTGAG	fwd		integration site determination, PCR1, HIV primer
MA.pr-529	GTAATACGACTCACTATAGGGC	rev		integration site determination, PCR1, linker primer
MA.pr-530	gcctccctcgcgccatcagCACTATAGGGCTCCGCTTAAGGGAC	fwd		integration site determination, PCR2, A-HIV primer
MA.pr-532	gccttgccagcccgctcagTCATGAGCAGACCCTTTTAGTCAGTGTGGAAAATC	rev		integration site determination, PCR2, B-barcode Mse-linker primer
MA.pr-534	gccttgccagcccgctcagCTACGATGAGACCCTTTTAGTCAGTGTGGAAAATC	rev		integration site determination, PCR2, B-barcode Nla-linker primer
fwd: forward primer; rev: reverse primer; RT: reverse transcription; PBS: primer binding site; psi: packaging signal;				
HMBS: hydroxymethylbilane synthase; PBGD: porphobilinogen deaminase; GAPDH: glyceraldehyde-3-phosphate dehydrogenase.				

**Table S4 : Gene expression assays**			
**Gene Symbol**	**ENSG_ID**	**Gene Name**	**AB Assay ID**
DZIP3	ENSG00000198919	DAZ interacting protein 3, zinc finger	Hs00978125_ml
GIMAP6	ENSG00000133561	GTPase, IMAP family member 6	Hs00226776_ml
GNG2	ENSG00000186469	guanine nucleotide binding protein (G protein), gamma 2	Hs00828232_ml
GRIP1	ENSG00000155974	glutamate receptor interacting protein 1	Hs00402711_ml
HDGF	ENSG00000143321	hepatoma-derived growth factor	Hs00610314_ml
HSP90AB1	ENSG00000096384	heat shock protein 90kDa alpha (cytosolic), class B member 1	Hs01546474_g1
IFI16	ENSG00000163565	interferon, gamma-inducible protein 16	Hs00194261_ml
IGSF3	ENSG00000143061	immunoglobulin superfamily, member 3	Hs00155437_ml
LY96	ENSG00000154589	lymphocyte antigen 96	Hs01026734_ml
PIGS	ENSG00000087111	phosphatidylinositol glycan anchor biosynthesis, class S	Hs00264209_ml
RCBTB2	ENSG00000136161	regulator of chromosome condensation (RCC1) and BTB (POZ) domain containing protein 2	Hs01048815_ml
RPL31	ENSG00000071082	ribosomal protein L31	Hs01015497_g1
RPS9	ENSG00000170889	ribosomal protein S9	Hs02339424_g1
SLC7A5	ENSG00000103257	solute carrier family 7 (amino acid transporter light chain, L system), member 5	Hs00185826_ml
SPON2	ENSG00000159674	spondin 2, extracellular matrix protein	Hs00202813_ml
